# A Service-Constrained Two-Stage Stochastic Programming Framework for Hospital Pharmacy Medication Supply Under Demand Uncertainty: Heterogeneous Demand, Conditional GARMA Scenarios, and Longitudinal Validation

**DOI:** 10.3390/healthcare14193135

**Published:** 2026-09-22

**Authors:** Fernando Rojas, Luis Arturo Muñoz-Aguirre

**Affiliations:** 1 Programa de Magíster en Gestión Farmacéutica y Farmacia Asistencial, Escuela de Química y Farmacia, Facultad de Farmacia, Universidad de Valparaíso, Valparaíso 2361845, Chile; luis.munozag@postgrado.uv.cl; 2 Centro de Micro-Bioinnovación, Escuela de Nutrición y Dietética, Facultad de Farmacia, Universidad de Valparaíso, Valparaíso 2361845, Chile; 3 Centro de Estudios del Comportamiento Alimentario, Escuela de Nutrición y Dietética, Facultad de Farmacia, Universidad de Valparaíso, Valparaíso 2361845, Chile; 4 Unidad de Farmacia, Hospital del Salvador, Santiago 7500922, Chile

**Keywords:** hospital pharmacy, stochastic programming, GARMA, temporal dependence, sample average approximation, scenario calibration, medication supply, intermittent demand, rolling-origin evaluation, service level

## Abstract

**Background/Objectives:** Hospital pharmacies must maintain medication availability despite heterogeneous and time-varying demand. Statistical fit alone may not yield well-calibrated future scenarios or the best replenishment decision. This study prospectively evaluates a decision-support framework combining probabilistic demand modelling, conditional temporal adaptation, explicit service requirements, and stochastic inventory optimization. **Methods:** Forty-eight monthly requested-demand observations for 13 anonymized medicines from a Chilean public hospital were evaluated through 12 expanding-window origins, yielding 156 prospective medicine-month decisions. A focused contextual consultation with three pharmacist experts was added to interpret the observed growth, decline, and intermittency patterns. Recurrent-positive and intermittent demand were modelled using normal, gamma, negative binomial type II (NBII), zero-inflated NBII (ZINBI), and hurdle negative-binomial models. When training data indicated trend or serial dependence, time-trend generalized linear models (GLMs) and generalized autoregressive moving average (GARMA) models were additionally considered as conditional scenario generators. The selected model supplied scenarios to a service-constrained two-stage stochastic inventory model with joint cycle-service and fill-rate requirements of 95% (SC95) and 99% (SC99). **Results:** Four of five recurrent-positive series favored the normal distribution; among intermittent series, seven showed numerical AIC ties between ZINBI and hurdle-NB and one favored normal. Four medicines showed positive temporal trends under the prespecified screen and two showed negative trends; eight had intermittent zero-request months, and M09 was both increasing and intermittent. Expert consultation indicated that growth and intermittency can arise from multiple clinical, organizational, inventory, and supply mechanisms rather than representing mutually exclusive demand types. A temporal model ultimately replaced the marginal generator in 46 of 156 origins, including GARMA in 19. Temporal adaptation increased central 90% held-out coverage from 84.0% to 88.5% overall and from 73.3% to 83.3% for recurrent-positive demand. SC95 achieved realized fill rate 0.991 and cycle service 0.929, whereas SC99 achieved 0.997 and 0.962, respectively. Scenario-expected versus realized cost MAPE was 2.4% and 0.7%, respectively. In a separate 900-case diagnostic simulation, 74.0% of cases were operational ties and AIC agreed with the operational winner in 13.7% of unique-winner cases. **Conclusions:** Hospital pharmacy replenishment should not rely on a single probability model or on statistical fit alone. Growth or decline in temporal direction and intermittency in demand occurrence should be interpreted as complementary dimensions, with pharmacist context used to avoid assigning unsupported causal meaning to anonymized series. Distributional heterogeneity and temporal dependence should guide scenario generation, while explicit service requirements and prospective validation should govern their translation into replenishment decisions.

## 1. Introduction

Medication supply in hospital pharmacies is a high-stakes operational process because replenishment decisions must be made before future demand, stockout risk, and procurement conditions are fully observed [1]. Shortages may compromise patient-care activities, increase the workload required to manage supply disruptions, and force emergency procurement or therapeutic substitution, whereas excessive inventory immobilizes scarce resources and increases storage, expiration, and obsolescence risks [2]. For this reason, hospital medication inventory should be framed as a decision-making problem under uncertainty, where replenishment policies must balance purchasing costs, holding costs, safety-stock requirements, shortage risk, and service-level requirements in settings characterized by variable demand and constrained resources [3,4,5,6].

The contemporary context reinforces this long-standing operational problem. The American Society of Health-System Pharmacists reported 227 active shortages in June 2026 and emphasized the additional workload created when shortages require changes to pharmacy automation and electronic health records [7]. These figures describe the United States and are not transferred numerically to the Chilean hospital studied here; they are used only as a recent external indicator of the continuing relevance of medication availability. Recent syntheses likewise recommend that hospital supply-chain performance be evaluated across financial, managerial, operational, and clinical domains rather than through expenditure alone [8], while pharmacy-inventory reviews continue to identify demand heterogeneity, information constraints, procurement rules, and institutional capacity as practical determinants of inventory performance [9].

Hospital pharmacy managers have traditionally used practical classification and replenishment tools to organize inventory decisions. These include ABC analysis, where items are grouped according to their contribution to annual consumption value or expenditure, and Vital, Essential, and Non-essential/Desirable (VEN/VED) matrices, where medicines are classified according to clinical criticality [10,11,12]. ABC and VEN/VED answer different managerial questions: the first is fundamentally economic, while the second is related to the consequences of unavailability. Their joint use can improve prioritization, but neither classification by itself identifies the probability distribution that represents future requested demand or the service target that should govern replenishment [13,14,15].

Hospital medication demand is heterogeneous in ways that are directly relevant to probabilistic modelling. Some medicines are requested every month and fluctuate around a relatively stable central level; others are strongly right-skewed, overdispersed, or characterized by repeated zero-request months. The distinction matters because a distributional assumption determines the probability assigned to low, central, and upper-tail demand and therefore influences any order-up-to quantity derived from that distribution. In intermittent-demand settings, occurrence and positive-demand size may carry different information, and methods that ignore this separation can produce misleading forecasts or inventory decisions [16,17,18,19,20].

For interpretation, this study distinguishes two complementary dimensions of demand behavior. The occurrence dimension classifies a series as recurrent-positive or intermittent according to whether zero-request months occur, whereas temporal direction describes increasing, stable, or decreasing trajectories. These dimensions are not mutually exclusive: an intermittent medicine may simultaneously exhibit a positive or negative temporal trend.

Although previous studies have advanced medication inventory optimization under shortage risk [4,5,6], multicriteria inventory management [13,15], simulation-based inventory policies [21], digital decision-support and medication-management technologies [22,23], and stochastic inventory modelling under uncertain demand [24,25], limited evidence integrates these perspectives into a single empirically testable workflow. The key unresolved issue is not whether sophisticated probability models can be fitted, but whether the information used to select them translates into better decisions when the next demand observation is genuinely unknown.

### 1.1. Problem Statement

Hospital pharmacies face a structurally asymmetric replenishment problem. Ordering too little can generate stockouts, emergency procurement, therapeutic substitution, and interruptions to pharmacy work, whereas ordering too much increases inventory exposure, storage requirements, expiration risk, and working-capital use. Because future medication demand is uncertain and the full clinical and organizational consequence of unavailability is rarely observable in administrative cost data, a purely monetary shortage penalty may understate the protection that replenishment should provide [26,27,28].

A second source of uncertainty concerns the demand model itself. Hospital portfolios combine recurrent-positive series with intermittent and zero-heavy demand, and some medicines also exhibit trend or serial dependence. A marginal distribution may represent the unconditional shape of demand adequately while missing temporal information needed to calibrate the next-period upper tail. Conversely, a dynamic model can add unnecessary complexity when the training history contains no meaningful temporal signal. The operational problem is therefore not simply to choose the distribution with the lowest information criterion, but to determine when demand-profile information and temporal structure materially improve scenario generation for a replenishment decision.

The resulting decision problem has three linked layers: statistical representation of heterogeneous demand, generation and validation of prospective demand scenarios, and stochastic inventory optimization under explicit minimum service requirements. Treating these layers separately allows statistical fit, scenario calibration, and operational value to be tested rather than assumed to coincide.

### 1.2. Study Objective and Contributions

The objective of this study is to develop and prospectively evaluate a hospital pharmacy decision-support framework that adapts the scenario-generating model to both the observed demand profile and its temporal structure before solving a service-constrained two-stage stochastic inventory problem. The framework is intended for settings in which longitudinal demand histories are routinely available, but complete inventory-state trajectories and fully monetized shortage consequences are not. By linking probabilistic demand representation, conditional temporal modelling, scenario generation, stochastic optimization, and out-of-sample validation within a single workflow, the approach seeks to bridge statistical model selection and operational replenishment decisions.

The study contributes in four connected ways. First, it compares continuous, overdispersed count, zero-inflated, and hurdle probability distributions across recurrent-positive and intermittent requested-demand profiles without inferring expenditure or clinical criticality from demand magnitude. Second, it introduces a prespecified temporal-escalation rule based exclusively on information available at each training origin. When the training data indicate relevant temporal structure, a parsimonious Gaussian generalized linear model (GLM) with a time trend and generalized autoregressive moving average (GARMA) models of orders GARMA(1, 0) and GARMA(2, 0) are allowed to compete with the marginal scenario generator. GARMA models extend generalized linear modelling by explicitly representing serial dependence in the conditional response and have previously been used to generate time-dependent pharmaceutical demand scenarios for stochastic-programming decisions [29,30]. Candidate marginal models are assessed using the Akaike information criterion (AIC) [31], whereas temporal candidates are compared using its small-sample corrected form, the corrected Akaike information criterion (AICc), to reduce unnecessary dynamic complexity in the 36–47-observation rolling windows [32].

Third, the model selected at each decision origin is used as a scenario-generating mechanism for a two-stage stochastic program solved through sample average approximation (SAA) [25]. Regular procurement is determined before demand realization as the first-stage decision, whereas scenario-specific shortage and overstock constitute second-stage recourse. Operational feasibility is imposed through simultaneous minimum cycle-service and fill-rate requirements before decision-sensitive cost is minimized. Fourth, repeated held-out months evaluate not only realized cost and service but also calibration of the generated demand scenarios. This prospective design distinguishes three related but non-equivalent dimensions of performance—statistical fit, out-of-sample scenario calibration, and operational decision quality.

The intended output is not a universal purchasing rule or a universally preferred forecasting family. It is an auditable operations framework in which model complexity is activated conditionally, service requirements are governed explicitly, and every replenishment recommendation can be evaluated against a subsequent unseen demand realization.

### 1.3. Conceptual Framework, Research Questions, and Hypotheses

The conceptual framework follows a chain from observed demand characteristics to probabilistic representation, from probabilistic representation to scenario generation, from scenarios to a service-constrained first-stage target, and from that target to realized operational consequences. Historical requested demand provides observable information on level, dispersion, zero share, trend, seasonality, and serial structure. Marginal and, when warranted, conditional temporal models translate that information into predictive scenarios. The two-stage decision layer then minimizes decision-sensitive cost only among targets that satisfy prespecified service constraints. Once the next requested-demand value is observed, scenario coverage, shortage, overstock, fill rate, cycle service, and cost can be evaluated prospectively.

Three research questions guide the study. RQ1: How do requested-demand profiles influence the statistical fit of alternative marginal probability models? RQ2: When pre-decision diagnostics indicate temporal structure, does a conditional GLM/GARMA branch improve prospective scenario calibration relative to marginal-only modelling, and does that improvement propagate to stochastic-programming cost and service outcomes? RQ3: Across broader randomized demand and cost conditions, how often do statistical-fit and operational winners agree? Five hypotheses are evaluated: recurrent-positive demand will more often favor lower-complexity marginal distributions (H1); intermittent demand will more often favor zero-mixture or hurdle models (H2); conditional temporal modelling will improve held-out scenario calibration at origins where temporal structure is detected (H3); explicit service constraints will materially improve realized availability relative to the unconstrained two-stage diagnostic solution (H4); and statistical-fit and operational winners will frequently diverge (H5).

The framework remains conditional rather than prescriptive. No distribution or temporal model is assumed to be universally appropriate, and the 95/95 and 99/99 service settings are analytical reference scenarios rather than universal clinical standards. Medicine-level criticality and supply conditions must govern the final service requirement.

## 2. Related Literature

Hospital medication inventory management combines several research traditions: item classification, stochastic inventory control, service-level design, probabilistic demand modelling, intermittent-demand forecasting, and decision support. The contribution of the present study lies at the intersection of these streams, with particular emphasis on how a fitted demand distribution should be translated into an operationally admissible replenishment target.

A first stream concerns classification and prioritization. ABC analysis and VEN/VED matrices remain widely used because they organize managerial attention according to expenditure and clinical criticality [10,11,12]. Multicriteria approaches extend these schemes by recognizing that expenditure, substitutability, shortage consequences, and other operational attributes cannot always be reduced to one ranking criterion [13,15]. These approaches are highly useful for deciding *which* items warrant differentiated control, but they do not by themselves specify the probability distribution of uncertain demand or the inventory required to achieve a target service level.

A second stream concerns the cost–service trade-off in inventory control. Classical inventory theory distinguishes service measures such as the probability of completing a replenishment cycle without shortage and the fill rate, which measures the proportion of demand supplied directly from available stock [14]. Cheng et al. show that cycle service and fill rate are distinct operational criteria and can lead to different inventory policies when cost and service are optimized jointly [33]. This distinction is particularly important in healthcare because shortage costs are difficult to monetize completely. Kelle et al. formulate hospital pharmacy inventory decisions using a minimum probability-of-no-shortage constraint, explicitly noting the difficulty of assigning shortage costs [26]. Neve and Schmidt similarly formulate a hospital point-of-use inventory model that minimizes cost subject to a minimum fill-rate requirement [28]. Pharmaceutical supply-chain models have also treated target service as an explicit constraint while minimizing total inventory cost [27]. Together, these studies provide a strong basis for treating service as a constraint on admissible replenishment decisions rather than as an outcome evaluated only after cost minimization.

A third stream addresses hospital inventory optimization and supply reliability. Simulation optimization has been applied to pharmacy settings to identify stock parameters under uncertain demand [21]. Other studies show that availability depends not only on local demand but also on disruption frequency, transshipment possibilities, recovery time, and supply redundancy [4,5,6]. These contributions demonstrate the operational importance of explicit shortage protection, but most begin with a selected demand representation or inventory policy rather than asking how heterogeneous demand characteristics should inform probabilistic model selection.

A fourth stream concerns probabilistic demand modelling. Distributional assumptions affect both scenario generation and the upper quantiles used for safety-stock or order-up-to decisions. Normal approximations are parsimonious for recurrent, relatively symmetric demand but permit negative support [34]. Gamma distributions provide positive, right-skewed support [35]. Negative-binomial models accommodate overdispersed count outcomes [36,37]. Hospital pharmacy lot-sizing research has shown that skewness and distributional form can materially alter inventory decisions under uncertainty [24]. The implication is that distribution choice is not only a descriptive question: it can determine how much inventory is required to satisfy the same service constraint.

A closely related operations-research contribution is the two-stage stochastic lot-sizing model of Rojas et al. [30]. That study used a GARMA representation to generate time-dependent pharmaceutical demand scenarios, selected temporal specifications with AIC, BIC, and deviance, and assessed the resulting stochastic-programming decisions out of sample. Its empirical case identified GARMA(2, 0) and showed that modelling temporal dependence improved scenario and lot-sizing accuracy relative to a time-independent GLM. The present study extends that logic from a single-product time-dependent illustration to a heterogeneous portfolio: marginal families first represent recurrent and zero-prone demand, while a prespecified training-only temporal screen conditionally activates Gaussian trend/GARMA candidates. The temporal model is therefore neither ignored nor imposed universally, and its incremental value is measured directly through held-out scenario coverage and downstream stochastic-programming outcomes.

A fifth stream comes from intermittent-demand forecasting. Intermittent and lumpy series contain irregular zero-demand periods and variable positive sizes, which can make conventional forecasting methods inadequate [16,17]. Occurrence–size formulations separate whether demand occurs from the magnitude of positive demand [18], while feature-based methods suggest that observable series characteristics can guide method selection [20]. Inventory consequences are central because forecast errors have asymmetric effects when stockout and obsolescence risks coexist [19]. Zero-inflated models and hurdle models offer alternative statistical representations of repeated zeros: the former uses a mixture mechanism, whereas the latter separates zero occurrence from a zero-truncated positive count process [38,39,40]. Neither formulation should be interpreted as identifying a causal zero-generating state unless the underlying operational mechanism is observed.

A final stream concerns pharmacy decision support. Clinical decision-support systems and medication-management automation illustrate the broader shift toward structured, auditable analytics in pharmacy practice [22,23]. For inventory applications, auditability requires more than reporting a winning distribution: decision makers should be able to trace the demand window, the fitted model, the service requirement, the resulting target, and the prospective consequence of that target.

Table 1 positions the proposed framework relative to these streams. The principal gap is not a lack of inventory models or forecasting methods but the limited integration of demand-profile-informed probabilistic selection with explicit service constraints and repeated prospective decision validation in a hospital pharmacy setting.

The novelty therefore has four components. First, demand-profile information determines model eligibility without conflating demand magnitude with expenditure or clinical criticality. Second, temporal complexity is introduced conditionally: trend and serial-dependence diagnostics are computed only on the training window and GARMA competes only when the evidence warrants it. Third, the chosen scenario model is translated into a service-constrained two-stage stochastic program rather than directly into an unconstrained cost target. Fourth, repeated unseen months evaluate statistical fit, probabilistic scenario calibration, expected-versus-realized stochastic cost, and realized service as distinct layers of evidence.

## 3. Materials and Methods

### 3.1. Design, Setting, and Demand Definition

This study uses two complementary analytical layers. The empirical layer evaluates real medicine-level requested demand from a Chilean public hospital, while the simulation layer examines model behavior across randomized demand and cost conditions. The empirical layer provides institutional realism; the simulation layer broadens the range of distributions, variability, intermittency, and cost exposure beyond the 13 observed series [21,24].

The empirical dataset contains 48 consecutive monthly observations for 13 anonymized medicines. The outcome is *requested demand*: quantities requested from the logistics unit, not purchases, dispensing, patient consumption, or stock-movement-derived consumption. A recorded zero is therefore not mechanically created by a pharmacy stockout, although it may still reflect institutional ordering and recording practices. For this reason, zero-inflated and hurdle models are interpreted as statistical alternatives rather than evidence of a specific causal zero state.

Demand heterogeneity is described using average monthly demand, standard deviation, coefficient of variation, zero-request frequency, and temporal indicators. Medicines with positive requested demand in every month are classified as recurrent-positive; medicines with at least one zero-request month are classified as intermittent. ABC expenditure and VEN/VED criticality are kept conceptually separate because neither can be inferred from demand magnitude alone.

The monthly time scale is an explicit design limitation. Monthly records align with the available administrative series and permit a 12-origin longitudinal evaluation, but they can conceal shorter stockout episodes, emergency purchases, delivery timing, and within-month demand dynamics. Demand profile is therefore interpreted at the resolution of the available requested-demand record, not as an intrinsic clinical property of a medicine.

Temporal structure is handled through a prespecified adaptive branch rather than as a post hoc warning. At every rolling origin, trend and serial-dependence diagnostics are calculated using only the training window. When these diagnostics indicate that a Gaussian conditional response is plausible and temporal structure is present, parsimonious time-trend GLM and GARMA candidates compete with the static marginal representation. This permits temporal dependence to improve scenario generation when supported by the data while limiting parameter proliferation in 36–47-observation training windows. Seasonality is examined separately because the 48-month series provide only four annual cycles: a descriptive joint F-test for 12-month sine/cosine terms after a linear trend is reported with the demand-profile results, while seasonal-naive demand from 12 months earlier is retained as the prospective seasonal benchmark. No unrestricted seasonal parametric branch is fitted.

#### Expert Contextual Consultation

To strengthen face validity and contextual interpretation of the observed demand trajectories, a focused consultation was conducted with three pharmacist experts familiar with hospital medication-use and supply processes. Open-ended questions addressed potential explanations for sustained increases and decreases in requested demand, months with zero requests, the possible role of seasonality in intermittent demand, illustrative medication classes, and the relationship between logistics requests and underlying clinical need. Responses were synthesized descriptively and used only to contextualize the quantitative findings. They were not used to relabel the anonymized medicines, alter model selection, or infer causality for individual M01–M13 series.

Figure 1 separates profiling, distribution fitting, service-constrained target construction, prospective evaluation, and simulation. This separation is essential because in-sample fit, one-step prediction, and operational acceptability are distinct evidence layers.

### 3.2. Operational Inputs and Cost Interpretation

Inventory decisions require a transparent mapping from quantities to consequences. In a conventional one-period representation, relevant components may include a fixed ordering cost, unit acquisition cost, holding cost for excess stock, and a shortage or emergency-procurement term. The relative magnitude of these components determines the economic critical fractile and therefore the balance between pre-positioning inventory and tolerating later shortage exposure [14,25]. Clinical and institutional consequences may be much broader than the accounting terms available in a dataset, so economic inputs should not be interpreted as complete measures of patient harm or organizational value.

Table 2 reports every operational input available in the study. The fixed ordering cost was CLP 20,312.65. The source costing exercise estimated holding cost as CLP 14.32 per unit per year; the revised monthly analysis therefore uses $h_{m}=14.32/12=1.1933$ CLP per unit-month. The shortage input is based on an 87.14% mean price differential between CENABAST and alternative procurement. To avoid presenting the premium as the full value of unmet demand, the cost function uses an emergency-procurement multiplier λ=1.8714, comprising ordinary acquisition plus the 87.14% premium. Monetary results are expressed in nominal 2023 CLP and are model-based decision-sensitive costs, not audited cash savings.

The available item-level data include unit acquisition prices and historical operational fields that document the economic scale of the medicines. However, a longitudinal inventory reconstruction requires month-specific opening stock, pending orders, lead times, deliveries, pack-size constraints, expiry dates, and potentially budget states. Those fields were not available consistently for all 48 months.

Initial stock, observed order, and the item-specific historical monetary-capacity field are available for December 2023 only. They are retained in Table 2 for source documentation and are not used as longitudinal constraints or imputed for earlier months. The principal longitudinal comparison therefore evaluates standardized order-up-to targets. This preserves prospective comparability without fabricating monthly inventory positions, deliveries in transit, pack sizes, or dynamic budget states.

### 3.3. Candidate Distributions

The candidate family was selected to represent distinct support and dispersion assumptions rather than to create a hierarchy in which complexity is always preferred. The normal distribution provides a parsimonious symmetric benchmark and is often used when aggregated demand is sufficiently large and stable [34]. Its limitation is that it is defined on the full real line, which can be inappropriate for low-volume or highly skewed non-negative demand.

The gamma distribution is continuous, non-negative, and right-skewed, and has long been considered in inventory-demand modelling [35]. It is particularly useful as a contrast to the normal model when positive demand is asymmetric. Gamma is treated as not applicable when the fitting window contains observed zeros, because a standard gamma model does not place mass at zero.

The negative binomial family is appropriate for non-negative count outcomes that exhibit overdispersion relative to a Poisson model. Different negative-binomial parameterizations exist, so the variance function must be stated explicitly [36,37]. The NBII form used here is defined by

(1) $$\mathrm{Var}\left(D\right)=\mu +\alpha \mu^{2},$$

where μ>0 and α>0. This is the common quadratic-variance NB2/NBII parameterization and is consistent with count-model and GAMLSS terminology [41].

Zero-inflated negative binomial modelling combines a zero-generating component with a negative-binomial count component [38,39]. Its flexibility can be useful when the observed zero frequency exceeds what an ordinary negative-binomial model represents, but a better statistical fit does not establish a particular causal origin of the zeros. For that reason, a hurdle-NB sensitivity model was added. The hurdle model estimates zero occurrence separately and uses a zero-truncated negative-binomial distribution for positive requested amounts [40]. Comparing NBII, ZINBI, and hurdle-NB helps distinguish whether improved fit comes from overdispersion alone, a mixture approximation, or explicit separation of occurrence and positive size.

Parameters were estimated by maximum likelihood and candidate models were compared by the Akaike information criterion (AIC) [31]. At each rolling origin, AIC was computed using only the fitting window available before the held-out month. AIC is interpreted as an in-sample information criterion, not as proof of superior prospective forecasting or operational value. For descriptive full-series reporting, two models whose AIC values differ by less than 0.01 are reported as a numerical tie rather than assigning an arbitrary winner. The rolling routine retains a deterministic minimum for reproducibility, but such near-ties are not interpreted as evidence of substantive superiority.

### 3.4. Conditional Temporal Scenario Models

The temporal branch is designed as a conditional escalation of the scenario generator, not as a second unrestricted model search. At each rolling origin, a linear time trend is tested on the training observations, and lag-2 Ljung–Box diagnostics are computed on the raw series and, when trend is present, on detrended residuals. A trend is considered operationally relevant at p<0.10. Autoregressive GARMA candidates are admitted only when residual serial dependence remains at p<0.10 after detrending, or when raw serial dependence is present in the absence of a significant trend. Detection of a temporal signal does not itself imply model replacement: the Gaussian temporal branch must also be eligible and a candidate must satisfy the AICc improvement rule described below. Annual seasonality is treated descriptively and through the seasonal-naive benchmark rather than through additional seasonal GARMA parameters because only four annual cycles are observed. All diagnostics precede the held-out observation.

Because the temporal branch uses a Gaussian conditional distribution, it is eligible for recurrent-positive series and for intermittent series only when the marginal AIC winner is normal. This allows an almost-recurrent intermittent series to enter the temporal comparison when its marginal behavior supports it, without forcing Gaussian dynamics onto strongly zero-inflated series. The conditional mean is written as

(2) $$\mu_{t}=\beta_{0}+\beta_{1}\tau_{t}+\sum_{h=1}^{p}\phi_{h}D_{t-h},\;D_{t}|H_{t-1}∼N\left(\mu_{t},\sigma^{2}\right),$$

where the time covariate $\tau_{t}$ is included only when the training-window trend test is significant. The candidate set comprises a Gaussian GLM with time trend and GARMA(1, 0) and GARMA(2, 0) identity-link specifications, with or without the time covariate as dictated by the screen. The autoregressive order is limited to p≤2 and no moving-average terms are fitted because each training window contains only 36–47 monthly observations. This parsimonious range is also consistent with the earlier hospital pharmaceutical application in which GARMA(2, 0) was selected [30].

Temporal candidates are compared on a common conditional sample beginning after the second lag. To reduce small-sample preference for additional parameters, model comparison uses the corrected Akaike Information Criterion,

(3) $$AIC_{c}=AIC+\frac{2k(k+1)}{n-k-1},$$

where *k* is the number of estimated parameters and *n* the conditional sample size [32]. A temporal candidate replaces the static Gaussian scenario generator only when its $AIC_{c}$ improves by at least two units. Otherwise, the marginal-AIC winner remains the scenario model. This rule makes the temporal branch conservative and fully reproducible.

For a selected temporal model, the one-step conditional mean and variance generate non-negative rounded demand scenarios for the next period. For a marginal model, scenarios are generated from its fitted unconditional distribution. The selected scenario generator then feeds exactly the same two-stage SAA and service constraints, ensuring that any downstream difference is attributable to the predictive scenario model rather than to a different inventory objective.

### 3.5. Service-Constrained Rolling-Origin Evaluation

The empirical evaluation reproduces the information sequence faced by a monthly decision maker. At origin *t*, observations through month *t* are available, candidate distributions are estimated, a target is selected before month t+1 is observed, and the target is evaluated only after requested demand for month t+1 becomes known. The expanding-window sequence begins with months 1–36 predicting month 37 and continues through months 1–47 predicting month 48. This produces 12 held-out decisions for each of 13 medicines, or 156 prospective medicine-month evaluations per policy.

The expanding window retains all earlier observations because the series are short and dispersion and zero frequency can be unstable in small samples. A fixed-width window could be preferable under strong non-stationarity, but its width would require an additional tuning decision that cannot be validated reliably with only 48 monthly observations. At every origin, all eligible marginal models are refitted and the AIC winner is identified using only the estimation window; the temporal screen described in Section 3.4 is then applied and may replace that marginal scenario generator with a qualifying GLM/GARMA model before any held-out demand is observed.

At each rolling origin and for each eligible fitted distribution, M=1024 predictive demand scenarios $D_{m}$ are generated with a fixed origin–medicine–model seed. These scenarios enter a two-stage sample-average approximation (SAA) [25,30]. The first-stage decision is the non-negative integer regular-procurement target *q*, selected before the next demand realization is observed. After scenario *m* is realized, shortage $S_{m}$ and overstock $O_{m}$ constitute second-stage recourse:

(4) $$\begin{array}{cc} O_{m} & \geq q-D_{m},\;O_{m}\geq 0, \end{array}$$

(5) $$\begin{array}{cc} S_{m} & \geq D_{m}-q,\;S_{m}\geq 0. \end{array}$$

With positive holding and emergency-procurement costs, these inequalities are tight at optimality, so $O_{m}={\left(q-D_{m}\right)}^{+}$ and $S_{m}={\left(D_{m}-q\right)}^{+}$. The finite-scenario stochastic program is

(6) $$\min_{q,\{O_{m},S_{m}\}}\;o\;⊮\left(q>0\right)+uq+\frac{1}{M}\sum_{m=1}^{M}\left(h_{m}O_{m}+\lambda uS_{m}\right),$$

where *o* is the fixed ordering cost, *u* the unit acquisition cost, $h_{m}$ the monthly holding cost, and λu the alternative emergency-acquisition cost. Equation (6) is a one-period two-stage recourse model embedded in each rolling origin; it is not presented as a reconstruction of a dynamic (s,S) system because monthly inventory position, pending orders, and lead times are unavailable longitudinally. The deterministic equivalent is one-dimensional and piecewise linear in *q*; the reproducibility code evaluates the scenario breakpoints and the minimum service-feasible integer target exactly, so no external MILP solver is required.

Two complementary service constraints are imposed on the same predictive scenario set. Scenario cycle service is

(7) $${\hat{\alpha }}_{M}\left(q\right)=\frac{1}{M}\sum_{m=1}^{M}⊮\left(S_{m}=0\right),$$

and scenario fill rate is

(8) $${\hat{\beta }}_{M}\left(q\right)=1-\frac{\sum_{m=1}^{M}S_{m}}{\sum_{m=1}^{M}D_{m}},$$

with admissibility requiring ${\hat{\alpha }}_{M}\left(q\right)\geq \alpha_{\min}$ and ${\hat{\beta }}_{M}\left(q\right)\geq \beta_{\min}$. Cycle service protects the frequency dimension of availability, whereas fill rate protects the volume dimension; neither measure dominates the other [14,26,28,33]. The service-qualified policy therefore minimizes expected decision-sensitive cost only within the set of targets satisfying both requirements.

Two prespecified scenarios are evaluated: SC95 with $\left(\alpha_{\min},\beta_{\min}\right)=\left(0.95,0.95\right)$ and SC99 with (0.99,0.99). These are analytical reference points rather than universal clinical standards. The appropriate threshold for a medicine should be assigned before optimization using clinical criticality, substitutability, supplier reliability, lead time, emergency sourcing options, and local governance [26,27,28].

For interpretation, the unconstrained SAA problem is also solved after removing both service constraints. In the special case with no fixed ordering charge and a continuous demand distribution, this one-period linear recourse model reduces to the classical newsvendor critical-fractile solution

(9) $$q_{NV}=F^{-1}\;\left(\frac{\lambda u-u}{\lambda u+h_{m}}\right).$$

The newsvendor quantity is therefore retained as a closed-form analytical reference for the unconstrained model, not as the primary algorithm used in the study. The fixed-charge SAA solution and the newsvendor reference are both stored in the reproducibility output. Because the observed emergency-price premium cannot represent the full clinical and organizational consequence of a shortage, the unconstrained stochastic solution is reported only as a diagnostic comparator and is not considered an admissible default hospital policy.

After the held-out monthly demand *d* becomes known, realized decision-sensitive cost is computed using the same accounting structure,

(10) $$C\left(q,d\right)=o\;⊮\left(q>0\right)+uq+h_{m}{\left(q-d\right)}^{+}+\lambda u{\left(d-q\right)}^{+},$$

together with realized shortage, overstock, fill rate, and cycle service. A nominal scenario constraint is therefore not assumed to guarantee the same realized service out of sample.

Scenario accuracy is evaluated twice at every rolling origin. First, the held-out demand is compared with the 5th and 95th percentiles of the minimum-AIC marginal model, providing a direct marginal-only baseline. Second, the same observation is compared with the temporal-adaptive scenario generator after the prespecified GLM/GARMA screen. We report central 90% predictive-interval coverage and the proportions below P05 and above P95 overall, by demand profile, and within temporal-branch origins. This follows the scenario-validation logic of Rojas et al. [30], while extending it across heterogeneous medicines and a conditional temporal-selection rule. To assess decision calibration, scenario-expected cost for SC95 and SC99 is compared with realized held-out cost using MAPE, Pearson correlation, and the proportion of decisions within ±10%.

The constraints are evaluated at the monthly decision horizon because this is the resolution of the available requested-demand data. In a deployed system with reliable lead-time information, the same logic should be applied to lead-time demand, and pack-size, shelf-life, storage, and budget constraints could be added to the feasible set without changing the two-stage service-first architecture.

Three transparent benchmarks are retained: rounded historical mean, previous-month requested demand, and seasonal-naive demand from 12 months earlier. They are used primarily to test whether probabilistic modelling adds predictive information beyond simple rules; they are not treated as service-qualified hospital policies unless they independently satisfy the same constraints. Outcomes include decision-sensitive cost, shortage and overstock units, realized fill rate, realized cycle service, mean absolute error (MAE), and mean absolute scaled error (MASE).

### 3.6. Randomized Simulation and Decision-Tree Validation

Simulation provides a controlled complement to a small empirical case. Its role is not to manufacture external validity but to examine whether a finding is sensitive to combinations of demand level, variability, intermittency, temporal change, and economic parameters that are difficult to observe repeatedly in one institution [21,25]. A fair simulation should vary the data-generating family rather than constructing conditions that mechanically favor the model whose behavior is under study.

The simulation contains 900 randomized cases. Each case draws a data-generating process from normal, gamma, NBII, ZINBI, or hurdle-NB; mean requested demand from a log-uniform range of 20–200,000 units; CV from 0.10 to 1.80; linear trend from −0.30 to 0.30 over the horizon; seasonal amplitude from 0 to 0.30; unit cost from CLP 10–17,000; and emergency multiplier from 1.2 to 4.0. ZINBI and hurdle cases draw a zero probability from 0.03–0.70, while normal, gamma, and NBII cases have no imposed structural-zero mechanism, although truncation of negative continuous draws can generate observed zeros. Forty-eight periods are used for fitting. For each eligible candidate fit, 256 predictive demand scenarios feed the same unconstrained two-stage SAA diagnostic, and the resulting target is evaluated over 12 new future periods. Thus, the empirical analysis uses 1024 scenarios per rolling decision, whereas this broader 900-case stress test uses 256 scenarios per candidate fit to keep the randomized experiment computationally tractable.

Statistical fit and operational performance are recorded separately. The statistical winner is the model with the lowest AIC on the fitting data. For every fitted model, the simulation stores mean realized cost over the 12 future periods, total shortage and overstock, demand-normalized shortage and overstock rates, and realized fill rate. The operational winner is the model with the minimum mean realized diagnostic cost; AIC-selected cost regret is measured relative to that within-case minimum. Because this simulation is intentionally service-unconstrained, its shortage and fill-rate outcomes are diagnostic consequences rather than recommended hospital service policies.

Operational results within 1% of the minimum are labeled ties. A classification tree is fitted only to cases with a unique operational winner. Winner classes occurring fewer than five times are retained in simulation summaries but excluded from tree fitting when independent stratified evaluation is not possible. The remaining data are separated into development and independent test samples; hyperparameters are selected within development using an internal validation split, the tree is refitted on all development data, and the test set is evaluated once [42]. The tree is therefore interpreted as an exploratory screening aid rather than a validated autonomous decision rule.

### 3.7. Reproducibility

Reproducibility is essential because the conclusions depend on repeated model fitting, simulation, and prospective decision calculations. The analysis package records the rolling origins, model estimates, held-out outcomes, simulation cases, tree validation split, generated tables, and figures.

The dataset uses anonymized identifiers M01–M13 and contains no patient-level information. A fixed random seed is used for simulation, and a verification script checks the number of rolling medicine-month evaluations, simulation cases, tables, figures, and independent tree-test observations. The versioned dataset and reproducibility materials are available through Zenodo at DOI https://doi.org/10.5281/zenodo.21456322 [43].

## 4. Results

### 4.1. Requested-Demand Profiles and Full-Series Fit

Figure 2 shows the 48-month series and highlights the rolling evaluation window. Table 3 reports continuous indicators rather than high/medium/low labels. Five medicines were recurrent-positive and eight intermittent. The observed zero share among intermittent series ranged from 0.021 to 0.688. Several series showed trend or serial dependence. The descriptive annual harmonic test identified nominal evidence of a 12-month component only for M12 (p=0.028); with only four annual cycles, this is treated as exploratory and the seasonal-naive rule remains the prospective seasonal benchmark.

Temporal direction and intermittency were not mutually exclusive. Four medicines met the prespecified p<0.10 temporal screen with positive trends (M01, M03, M04, and M09), whereas two showed negative trends (M05 and M06). M09 was both intermittent and increasing. The observed portfolio therefore included growing, declining, and stable trajectories as well as recurrent-positive and intermittent occurrence patterns.

The three pharmacist consultations provided convergent contextual explanations for these patterns. Increasing requested demand was associated with mechanisms such as new or expanded clinical indications, greater coverage or patient volume, earlier diagnosis, implementation of new services or programs, stronger adherence to protocols, therapeutic substitution, and centralization of treatments previously obtained through other channels. Decreasing demand was associated with discontinuation or replacement of therapies, safety or protocol changes, treatment optimization, lower administration frequency, reduced eligible caseload or disease incidence, and, in some circumstances, supply or budget constraints. Zero-request or intermittent months were linked not only to seasonality but also to low caseload, rare or episodic indications, advance purchasing or residual stock, temporary substitution, supply constraints, and administrative timing. Illustrative examples offered by the experts included direct oral anticoagulants, biologics, oncology medicines, and specialized therapies for increasing demand; therapies displaced by safer or more modern alternatives, including some antibiotics, for declining demand; and antidotes, orphan medicines, immunoglobulins, and specific coagulation factors for intermittent demand. The experts also emphasized that requested demand is an operational proxy for clinical need because inventory position, consolidated purchasing, budget, supplier availability, substitution, and replenishment policies can alter quantities requested without an equivalent change in underlying patient need. These examples are illustrative and are not mapped to the anonymized M01–M13 series.

Full-series AIC comparisons are shown in Table 4. Four of five recurrent-positive medicines favored normal and one favored gamma. Among the eight intermittent medicines, seven showed numerical AIC ties between ZINBI and hurdle-NB and one (M09) favored normal. The result therefore supports the relevance of zero-aware mixture or occurrence–size representations at the family level, but it does not identify ZINBI as superior to hurdle-NB in these short series. Figure 3 displays representative fitted shapes for recurrent-positive M03 and intermittent M13.

### 4.2. Conditional Temporal Selection and Scenario Calibration

The prespecified training-only screen detected a temporal signal in 82 of the 156 rolling origins, the Gaussian temporal branch was eligible in 72, and a temporal model ultimately replaced the marginal scenario generator in 46 (Table 5). Among those 46 selected temporal models, GARMA was chosen in 19 origins and a Gaussian time-trend GLM in 27. The pattern was medicine-specific rather than universal: M01 selected GARMA in twelve origins; M03 selected a trend GLM in eight origins and GARMA in four; M04 selected a trend GLM in ten; and M09 selected a trend GLM in nine and GARMA in three. Thus, detection, eligibility, and final temporal selection are distinct stages, and GARMA was retained only when it added sufficient small-sample information beyond the simpler static alternative.

Temporal adaptation materially improved prospective scenario calibration (Table 6; Figure 4). Across all 156 origins, central P05–P95 coverage increased from 84.0% for the minimum-AIC marginal model to 88.5% for the temporal-adaptive scenario generator, while upper-tail exceedance fell from 12.8% to 6.4%. The improvement was concentrated where temporal structure was a plausible source of misspecification. For recurrent-positive medicines, coverage increased from 73.3% to 83.3% and P95 exceedance fell from 20.0% to 8.3%; intermittent demand was already close to nominal and moved from 90.7% to 91.7%. Within the 46 temporal-branch origins alone, coverage increased from 65.2% to 80.4% and upper-tail exceedance fell from 34.8% to 13.0%.

Point accuracy improved in the same direction. Overall MAE declined from 3500.4 units under the marginal-only scenario model to 2782.0 under temporal adaptation, a 20.5% reduction. Within temporal-branch origins, MAE declined from 8023.5 to 5586.9 units, a 30.4% reduction. The largest calibration gain occurred for M03, where central-90% coverage increased from 16.7% to 66.7%, while M09 increased from 58.3% to 66.7%. These representative improvements are illustrated in Figure 4. They do not establish perfect calibration, but they show that upper-tail failures identified under marginal-only modelling were partly recoverable using information available before the held-out month.

### 4.3. Prospective Service-Constrained Performance

The temporal-adaptive scenario generator feeds the service-constrained two-stage policies summarized in Table 7. Across the 156 prospective medicine-month decisions, SC95 achieved realized mean fill rate 0.991 and cycle service 0.929 at mean decision-sensitive cost CLP 6.528 million per medicine-month. SC99 increased realized fill rate to 0.997 and cycle service to 0.962 at CLP 6.922 million. Aggregate shortage declined from 22,044 units under SC95 to 14,415 under SC99, while overstock increased from 1,025,119 to 1,389,641 units. Cost dispersion remained substantial across heterogeneous medicines and months, with standard deviations of CLP 11.120 million for SC95 and CLP 11.643 million for SC99. The resulting cost–service trade-off is shown in Figure 5.

The unconstrained two-stage diagnostic solution remained much less protective, with mean fill rate 0.749 and cycle service 0.532 at mean cost CLP 5.901 million. Relative to this cost-only diagnostic, SC95 increased mean decision-sensitive cost by 10.7% and SC99 by 17.3%. As illustrated in Figure 5, these differences are not interpreted as conventional efficiency losses because the unconstrained solution does not satisfy the prespecified hospital service requirements; rather, they quantify the incremental inventory consequence of explicit availability protection under the same accounting inputs.

The nominal-versus-realized distinction also improves after temporal adaptation. SC95 still fell short of the nominal 0.95 cycle-service target prospectively, achieving 0.929, while SC99 achieved 0.962. Realized fill rates were 0.991 and 0.997, respectively. The results therefore support both components of the framework: explicit service constraints are necessary, but improved scenario calibration is also needed if nominal service requirements are to translate reliably into held-out outcomes.

Medicine-level results in Table 8 show that aggregate service measures conceal substantial heterogeneity, reinforcing prospective item-level monitoring. The historical-mean, previous-month, and seasonal-naive rules remain useful predictive benchmarks in Table 7. Seasonal-naive demand achieved the lowest point-prediction MAE among the simple rules, reinforcing the importance of temporal information. The temporal-adaptive probabilistic framework is not justified by point prediction alone; its additional role is to provide conditional predictive scenarios that can support explicit tail-sensitive service constraints within the stochastic program.

The calibration results in Table 6 also show that improved demand-scenario representation propagated into stable stochastic-programming cost estimates. For SC95, scenario-expected versus realized cost had MAPE 2.4%, correlation 0.997, and 95.5% of medicine-months within ±10%. For SC99, the corresponding values were 0.7%, 0.999, and 97.4%. These results indicate that demand-scenario calibration and decision calibration are related but should be evaluated separately.

### 4.4. Simulation, Statistical–Operational Divergence, and Tree Performance

Of 900 randomized cases, 666 (74.0%) were operational ties within 1% and 234 had a unique operational winner. Among unique winners, the AIC and operational model agreed in 13.7%. Across all cases, the model selected by AIC had mean cost regret of 2.19% and median regret of 0.11% relative to the least-cost fitted model in the same case. Its mean diagnostic shortage rate was 0.564, overstock rate 0.317, and fill rate 0.436 (Table 9). These service-unconstrained outcomes are not hospital recommendations; they quantify the operational consequences of fit-based model choice in the diagnostic stress test. Each candidate fit used 256 SAA scenarios and was evaluated over 12 new periods (Figure 6).

Figure 7 reports the full fit–operation matrix. The exploratory tree used 163 development and 71 independent test cases; no unique-winner class required exclusion under the five-case rule. Test accuracy was 0.056 and balanced accuracy 0.213 (Table 10). This very weak independent performance is itself informative: simple demand and cost descriptors are insufficient to replace explicit stochastic optimization with a classification shortcut. The tree is retained as a negative screening result rather than promoted as a procurement rule (Figure 8).

## 5. Discussion

The main contribution of this study is not a universally superior demand distribution. It is an adaptive operations architecture that separates four decisions that are often conflated: which marginal family is statistically plausible, whether temporal dependence warrants a conditional scenario model, what minimum service the institution requires, and which service-feasible target minimizes decision-sensitive cost. This separation permits each layer to be validated prospectively.

The full-series marginal results confirm demand heterogeneity. Four of five recurrent-positive medicines favored normal, whereas seven of eight intermittent medicines produced numerical AIC ties between ZINBI and hurdle-NB and one favored normal. This remains consistent with intermittent-demand research emphasizing zero occurrence and irregular positive size [17,18]. However, the rolling temporal results add an important qualification: marginal shape is not the whole predictive problem. A recurrent-positive medicine can be approximately Gaussian marginally and still require conditional modelling of trend or serial dependence to represent the next-period upper tail.

This provides a direct extension of the pharmaceutical stochastic-programming evidence of Rojas et al. [30]. Their single-product study showed that GARMA-based time-dependent scenarios could improve scenario and lot-sizing accuracy relative to a time-independent GLM. The present portfolio analysis reaches a compatible but more nuanced conclusion. A temporal signal was detected in 82 of 156 prospective origins, the Gaussian branch was eligible in 72, and a temporal model ultimately replaced the marginal generator in 46. Among those 46 selections, GARMA was chosen in 19 and a simpler time-trend GLM in 27. Complexity therefore should not be imposed because temporal dependence is theoretically possible. It should be activated when pre-decision diagnostics and small-sample model selection demonstrate incremental information.

The scenario-calibration gains support H3. Overall central-90% coverage increased from 84.0% to 88.5%, and recurrent-positive coverage increased from 73.3% to 83.3%. More importantly for inventory protection, recurrent-positive observations above the predicted P95 fell from 20.0% to 8.3%. Within temporal-branch origins the improvement was larger, from 65.2% to 80.4% coverage and from 34.8% to 13.0% upper-tail exceedance. These results suggest that some apparent tail misspecification under marginal models was actually unmodelled temporal evolution. They also show the limit of the correction: coverage did not become perfect, so GARMA should be viewed as a conditional improvement, not a guarantee.

The result for M03 is particularly instructive. Its central-90% coverage increased from 16.7% under marginal-only modelling to 66.7% after temporal adaptation, while M09 improved from 58.3% to 66.7%. M09 is classified as intermittent but can enter the Gaussian temporal branch when its marginal winner is normal, illustrating why demand-profile labels should guide eligibility without rigidly determining the final model. The framework is therefore hierarchical rather than categorical: zero structure, marginal support, trend, and serial dependence contribute different information.

The expert consultation strengthens the face validity of this distinction. Pharmacists described increasing requested demand as potentially reflecting expanded indications, coverage, diagnosis, treatment programs, protocol adherence, substitution, or service centralization, whereas decreasing demand may follow therapeutic replacement, safety-related changes, lower treatment frequency, or reduced caseload. They also emphasized that intermittent zero-request months need not be seasonal: rare or episodic indications, existing stock, advance purchasing, substitution, supply disruptions, and administrative timing can all generate zeros. This interpretation is consistent with the quantitative seasonality diagnostic, which identified nominal evidence of a 12-month harmonic component only for M12. Because the medicines remain anonymized, these professional explanations provide contextual plausibility rather than medicine-specific causal attribution.

The operational consequence is visible in the two-stage results. SC95 now achieved realized fill rate 0.991 and cycle service 0.929, while SC99 achieved 0.997 and 0.962, respectively. The higher service scenario crossed the 0.95 realized cycle-service reference after temporal adaptation. At the same time, scenario-expected costs closely tracked realized costs, with MAPE 2.4% for SC95 and 0.7% for SC99. Scenario calibration and decision calibration are not identical, but both improved enough to support the proposition that a better scenario generator can strengthen the downstream stochastic program.

The more general operations insight is that model fit, scenario calibration, and decision quality form a chain rather than synonyms. AIC or AICc evaluates a model relative to its estimation sample; predictive-interval coverage asks whether the scenario distribution represents unseen demand; the two-stage program maps that distribution into a first-stage decision and second-stage recourse; realized cost and service then evaluate the operational consequence. Reporting all four layers prevents a statistically attractive model from being promoted directly into a purchasing rule.

The separate randomized simulation remains complementary. It deliberately studies marginal model fit versus unconstrained cost-loss consequences without the empirical temporal branch so that the statistical–operational divergence is not obscured by common high service floors. Its 74.0% operational-tie rate and 13.7% AIC–operation agreement among unique winners, together with mean AIC-selected cost regret of 2.19%, show that many distributional distinctions either have negligible decision impact or identify a different operational winner. This reinforces rather than contradicts the temporal result: added complexity requires evidence both that it improves scenarios and that the resulting decisions matter.

Taken together, the analyses provide an explicit assessment of H1–H5. H1 is supported because four of five recurrent-positive series favored the parsimonious normal family. H2 is supported at the level of zero-aware model structure rather than a single preferred distribution: seven intermittent series showed effectively indistinguishable ZINBI and hurdle-NB AIC values, while one favored normal. H3 is supported by improved held-out interval coverage after conditional temporal adaptation. H4 is supported by the substantial gain in realized availability under SC95 and SC99 relative to the unconstrained diagnostic solution. H5 is supported by the high operational-tie rate and low AIC–operational agreement in the randomized simulation. These findings also qualify the hypotheses: demand profile narrows plausible models, but neither complexity nor a small information-criterion advantage should be converted automatically into a purchasing rule.

### 5.1. Interpreting the Cost–Service Frontier

The empirical comparison should be read as a cost–service frontier rather than as a contest in which the least expensive policy necessarily wins. Moving from the unconstrained diagnostic target to SC95 substantially increases realized availability, and moving from SC95 to SC99 provides further protection at additional inventory cost. This is the expected structure of a service-constrained inventory problem: stronger availability requirements shrink the feasible set and generally require a larger order-up-to target. The relevant managerial question is therefore whether the incremental cost of moving along the frontier is justified by the clinical and operational consequences of shortage for the medicine under consideration.

This interpretation is especially important because fill rate and cycle service can move differently. Fill rate weights shortage by demand volume, whereas cycle service treats each medicine-month as a shortage or no-shortage event. A portfolio can therefore display a very high aggregate fill rate while still experiencing several small stockout events. Conversely, a policy can avoid shortage in most periods but perform poorly in the few periods in which demand is unusually large. Reporting both measures prevents a single aggregate service statistic from masking these different failure modes and is consistent with inventory-control research that treats Type-I and Type-II service as distinct criteria [14,33].

The observed SC95 result illustrates this distinction. Its realized fill rate of 0.991 indicates that most requested volume was covered, yet its cycle service of 0.929 shows that shortages still occurred in a nontrivial share of medicine-months. Rather than invalidating the constrained formulation, this gap reveals imperfect high-quantile calibration when short heterogeneous series are modelled with stationary marginal distributions. SC99 raises the nominal protection and achieves 0.997 realized fill rate and 0.962 cycle service, which is much closer to the intended no-shortage frequency. The empirical lesson is therefore that a nominal service requirement must be paired with prospective calibration monitoring; the nominal percentage should not be interpreted as an automatically achieved operational guarantee.

For hospital implementation, the frontier also creates a transparent basis for differentiated governance. A Vital medicine with no immediate substitute and a long or unreliable replenishment lead time may warrant operation near the high-protection end of the frontier, whereas a Desirable item with multiple substitutes and rapid emergency sourcing may tolerate a lower service target. The model does not infer this clinical preference from demand volume. Instead, it quantifies the inventory and cost consequences once the preference has been specified. This separation preserves the role of pharmacist judgment while making the quantitative consequence of that judgment reproducible and auditable.

### 5.2. Service-Level Governance and Operational Calibration

A key implication is that the service floor is a governance parameter rather than a parameter that should be estimated automatically from the demand series. Demand data can indicate how much inventory is required to pursue a given level of availability, but they cannot determine how clinically acceptable a shortage is. That judgment belongs to medication governance and should draw on criticality, therapeutic substitutability, supplier reliability, lead time, emergency sourcing, storage limitations, and institutional risk tolerance. This division of responsibilities is consistent with the logic of VEN/VED classification: clinical importance determines the consequence of unavailability, while probabilistic modelling estimates the protection required under uncertain demand [10,12].

The use of two service measures also creates an interpretable diagnostic for policy design. If the cycle-service constraint is binding while the fill-rate requirement is slack, the principal challenge is the probability of experiencing any shortage and the upper quantile of the predictive distribution drives the target. If the fill-rate requirement is binding, the cumulative expected magnitude of shortage is the limiting consideration. If neither service constraint changes the unconstrained SAA optimum, then the cost-minimizing solution already provides the specified protection under the information available at that origin. Recording which constraint is binding can therefore help pharmacists understand why a target increased and whether the increase is driven by event frequency, shortage volume, or both.

The empirical difference between SC95 and SC99 illustrates why calibration should be treated as a repeated operational process. The nominal 95/95 specification achieved a realized fill rate well above 0.95 but a realized cycle service below 0.95. Raising both nominal floors to 0.99 produced approximately 0.95 realized cycle service in this dataset, but that relationship should not be extrapolated mechanically to another hospital or medicine. Instead, a deployed system could maintain a rolling calibration record that compares nominal α and β targets with realized cycle service and fill rate. Persistent underachievement would trigger model review, a higher nominal floor, a shorter estimation window, or a different demand model; persistent overprotection could prompt review of excess inventory and the appropriateness of the service target.

This monitoring approach avoids false precision in the choice of 0.95 or 0.99. The values are useful because they define transparent reference points and are commonly examined in inventory service analyses, including hospital settings [28,33]. They should nevertheless be interpreted as policy scenarios. A clinically Vital medicine with no reliable substitute may justify a service floor above the values studied here, whereas a readily substitutable Desirable item may not. The framework can accommodate either decision because the service requirement is separated from distribution fitting and cost evaluation. What must remain constant is the governance rule that the threshold is selected before observing the next demand outcome and that any change in the threshold is documented.

A practical implementation could therefore maintain, for each medicine, four linked records: the demand-profile indicators and model diagnostics; the locally approved service floors and their clinical or supply rationale; the constrained target produced at each decision origin; and the subsequent realized service and inventory consequences. This creates an audit trail in which a statistical recommendation can be challenged without losing reproducibility. It also makes expert override explicit: a pharmacist may increase a target because of a known shortage, formulary change, or supplier warning that is absent from the historical demand series, but the override and its reason can be distinguished from the model-generated baseline. Such a design is more consistent with decision support than with autonomous purchasing.

### 5.3. Limitations and Future Research

The empirical evidence comes from one public hospital and 13 medicines, so external generalizability is limited. The simulation broadens the analytical conditions but does not create multicenter validation. Hospital formularies, procurement systems, supplier markets, clinical priorities, and data-recording practices vary substantially, and the service thresholds used here should not be transferred without local evaluation.

The requested-demand series are monthly and contain 48 observations per medicine. This is sufficient for the 12-origin expanding-window design but remains short for unrestricted dynamic model search and provides only four annual cycles. The temporal branch is therefore deliberately parsimonious: Gaussian identity-link GLM/GARMA models, autoregressive orders no greater than two, and no moving-average or seasonal terms. The annual harmonic test is descriptive rather than a basis for seasonal-model selection, while seasonal-naive demand provides a prospective benchmark. Dynamic gamma, negative-binomial, zero-inflated, hurdle, state-space, occurrence–size, and richer seasonal models should be evaluated with longer series.

A second limitation is incomplete longitudinal inventory state. Month-specific opening inventory, pending orders, delivery dates, lead times, pack sizes, shelf lives, and item-level budget constraints were not available consistently. These fields were not imputed. Consequently, the rolling analysis evaluates standardized order-up-to targets and decision-sensitive cost rather than reconstructing a complete dynamic inventory-control system.

The shortage parameter represents an emergency-procurement price differential and not the full social or clinical cost of unavailability. The service-constrained formulation is intended to reduce dependence on that incomplete valuation, but it does not determine the clinically correct service threshold. Both cycle-service and fill-rate constraints are evaluated on the same predictive SAA scenario set. Their realized out-of-sample performance therefore depends on scenario calibration, and neither nominal floor should be interpreted as a deterministic service guarantee.

SC95 achieved 0.929 realized cycle service despite a nominal 0.95 scenario floor, so residual prospective miscalibration remains an empirical limitation even after temporal adaptation. The branch improved upper-tail coverage substantially but did not eliminate all misses. Longer series, conformal or distribution-free calibration, dynamic occurrence–size models for zero-heavy demand, and explicit lead-time demand modelling may further improve the relationship between nominal and realized service. Future studies should also validate the p<0.10 temporal screen and the two-unit AICc improvement rule rather than treating them as universal thresholds.

The diagnostic simulation is intentionally unconstrained by service in order to isolate fit–cost divergence, and its data-generating processes do not include autoregressive GARMA dependence. Future simulation work should explicitly vary temporal dependence, trend breaks, correlated demand across medicines, clinically stratified service floors, formulary changes, supplier disruptions, alternative-procurement delays, and multi-period inventory carry-over. The 1% operational-equivalence band should likewise be varied in sensitivity analyses.

Future multicenter studies should prospectively record inventory position, lead time, pending supply, pack size, shelf life, VEN/VED status, substitution options, emergency purchases, realized service consequences, and supplier reliability. These data would permit a full dynamic model in which service floors are medicine-specific and validated against both operational and clinical outcomes.

The pharmacist consultation is also intentionally limited in scope. Three expert responses were used to strengthen contextual interpretation and face validity, not to achieve qualitative saturation or establish causal explanations for individual medicines. The illustrative medication classes supplied by the experts cannot be linked to M01–M13 because therapeutic identities remain anonymized. Future studies could prospectively pair medicine identity, prescribing indications, formulary changes, patient volume, stock position, and procurement events with the demand series to test the proposed mechanisms directly.

## 6. Conclusions

This study develops a hospital pharmacy operations framework in which heterogeneous marginal demand models and a conditionally selected temporal GLM/GARMA layer generate scenarios for a service-constrained two-stage stochastic program. Full-series results showed four normal and one gamma winner among recurrent-positive medicines, while seven of eight intermittent medicines produced numerical AIC ties between ZINBI and hurdle-NB, supporting zero-aware modelling without claiming superiority of one zero formulation.

Across 156 prospective origins, a temporal signal was detected in 82, the Gaussian temporal branch was eligible in 72, and a temporal model ultimately replaced the marginal generator in 46; GARMA was selected in 19 and a simpler trend GLM in 27. Temporal adaptation increased central-90% scenario coverage from 84.0% to 88.5% overall and from 73.3% to 83.3% for recurrent-positive demand. SC95 achieved a realized fill rate of 0.991 and a cycle service of 0.929, while SC99 achieved 0.997 and 0.962, respectively. Scenario-expected versus realized cost MAPE was 2.4% and 0.7%. In the separate 900-case diagnostic simulation, 74.0% of cases were operational ties and AIC agreed with the operational winner in 13.7% of unique-winner cases.

The practical implication is a staged governance sequence: characterize marginal demand; examine trend, seasonality, and serial dependence using only pre-decision information; generate scenarios with the simplest supported model; assign service floors according to clinical and supply criticality; solve the two-stage stochastic program; and monitor realized calibration, service, and cost. Statistical fit, scenario accuracy, and operational value should be reported as distinct evidence. Multicenter validation with richer inventory-state, lead-time, supplier, and medicine-criticality data is required before prescriptive deployment.

The added pharmacist consultation further clarifies that temporal growth or decline and intermittent occurrence are complementary features of requested demand rather than exclusive categories. Operational and clinical context is therefore necessary before attributing observed trends or zero-request months to patient need alone.

## Figures and Tables

**Figure 1:**
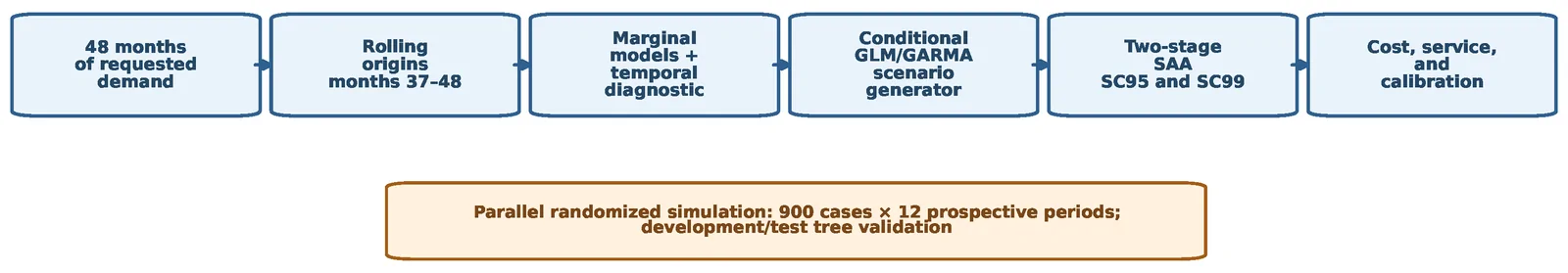
Empirical and simulation workflow. Every rolling-origin decision uses only information available before the evaluated month; service constraints are imposed before prospective cost and availability are assessed.

**Figure 2:**
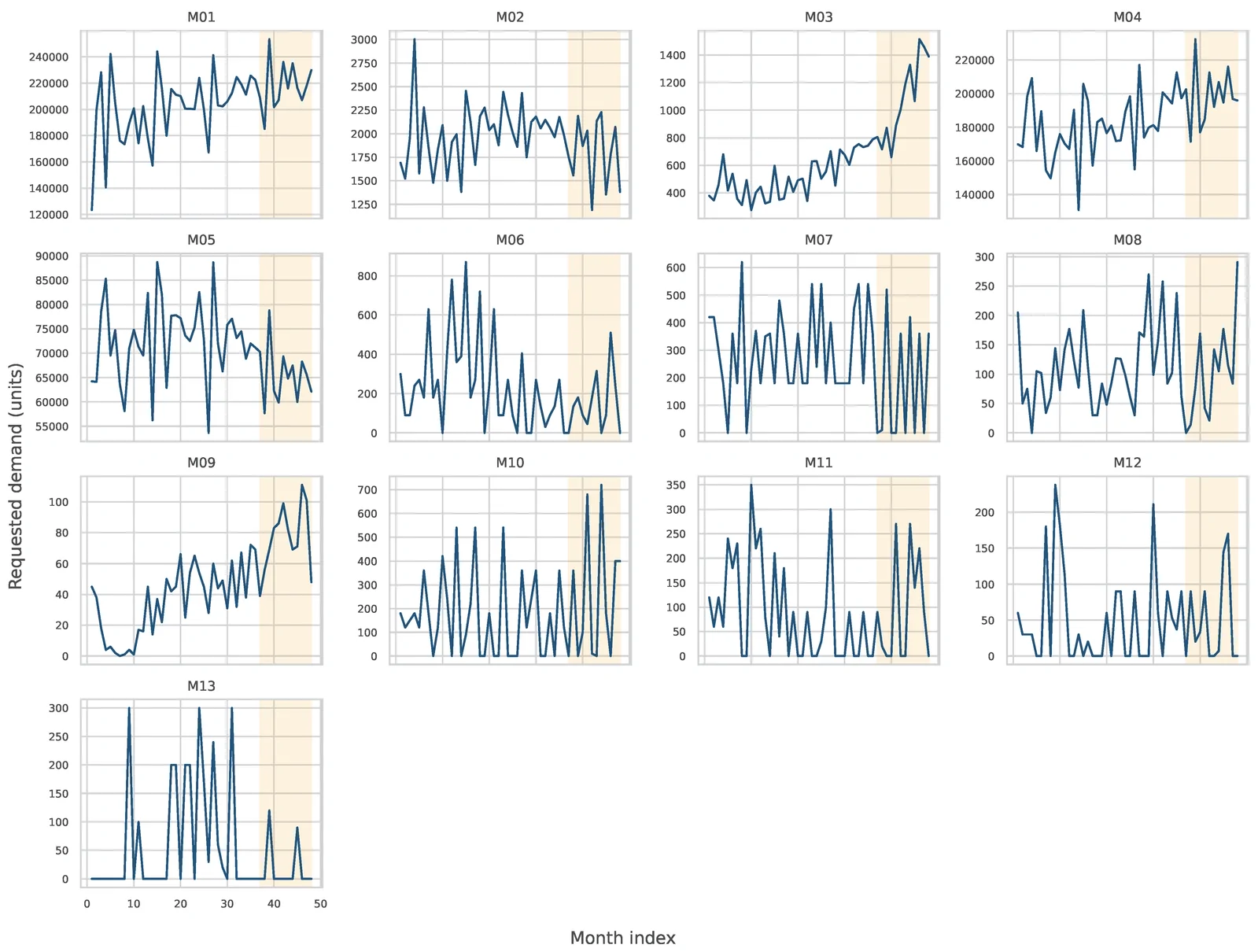
Monthly requested-demand profiles. The shaded interval (months 37–48) is evaluated through expanding-window origins.

**Figure 3:**
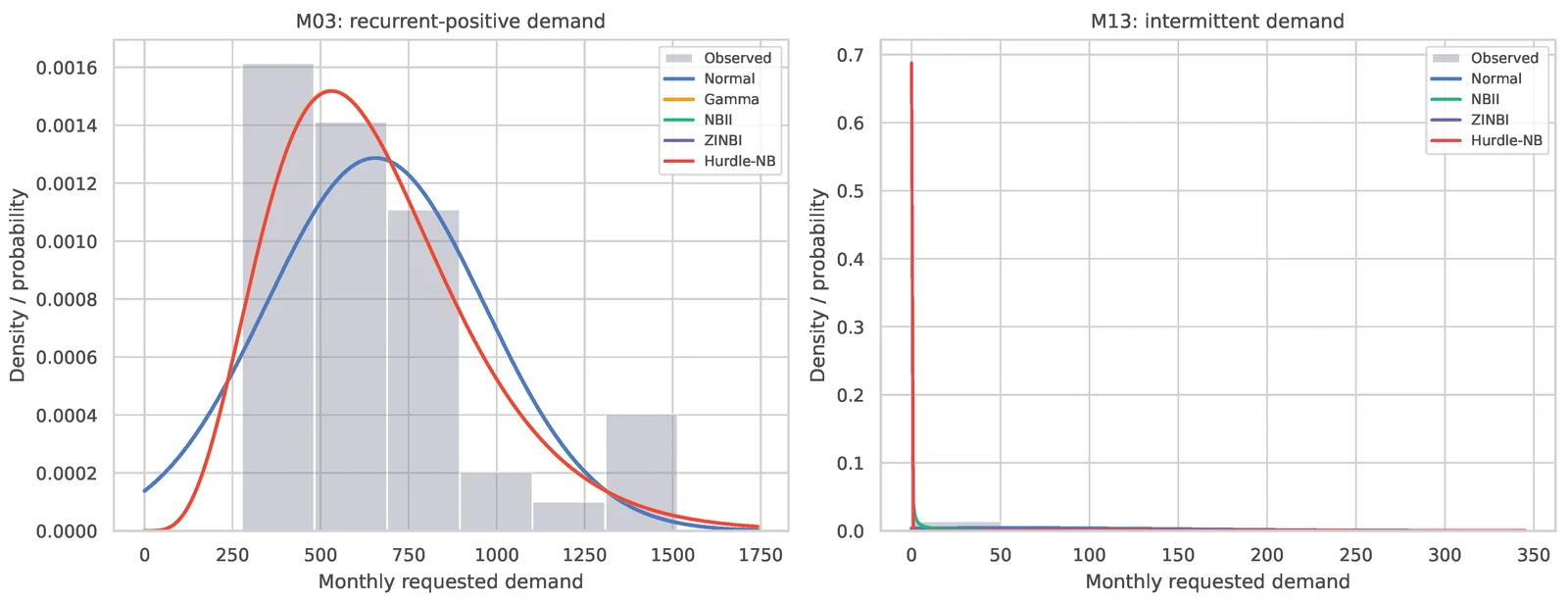
Representative observed requested demand and fitted distributions. The (**left**) panel illustrates recurrent-positive demand; the (**right**) panel illustrates an intermittent series with many zeros.

**Figure 4:**
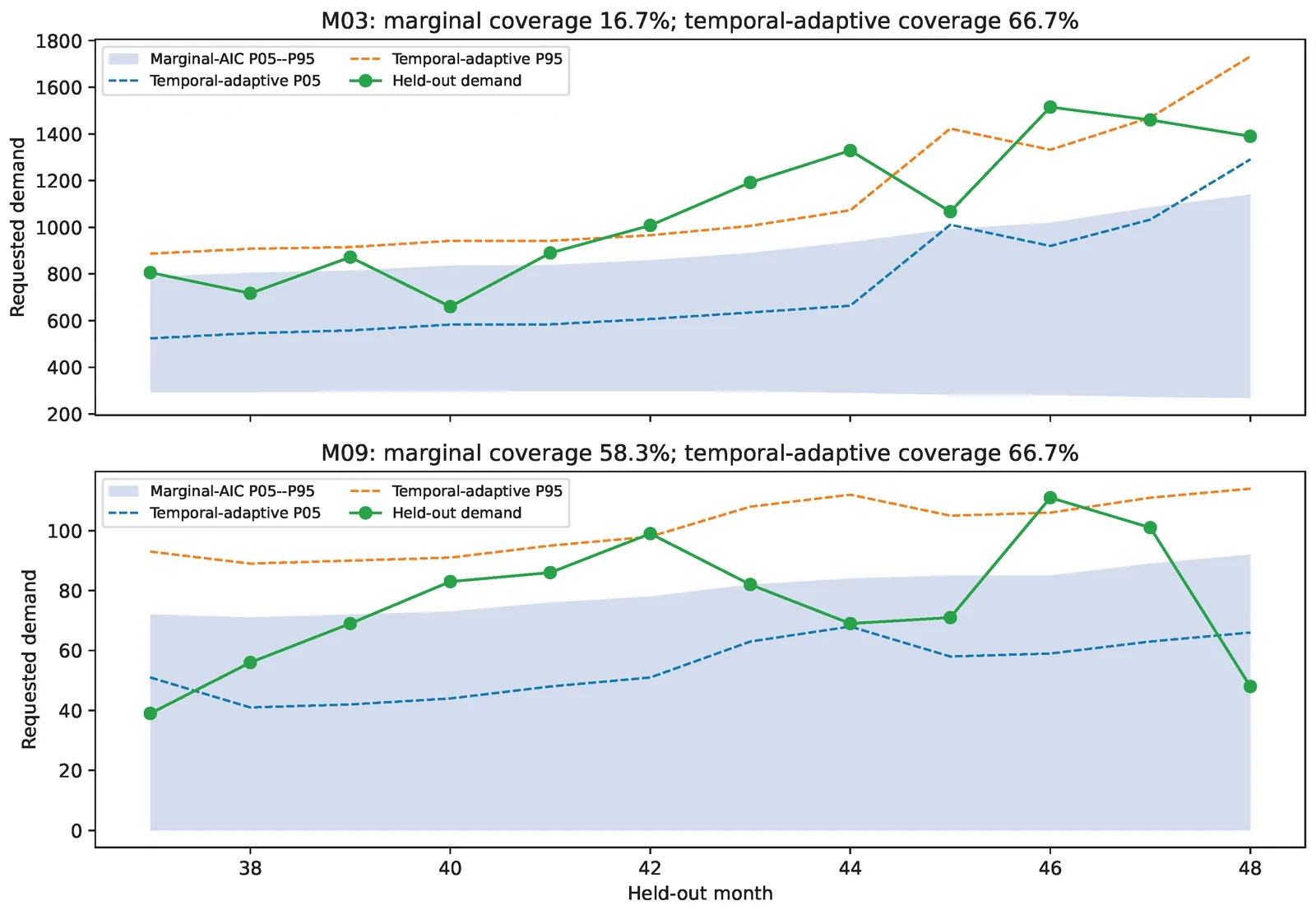
Prospective scenario calibration for representative medicines M03 and M09. Held-out requested demand is shown against central 90% intervals from the minimum-AIC marginal model and the temporal-adaptive GLM/GARMA scenario generator. Every temporal decision and interval is based only on the corresponding training window.

**Figure 5:**
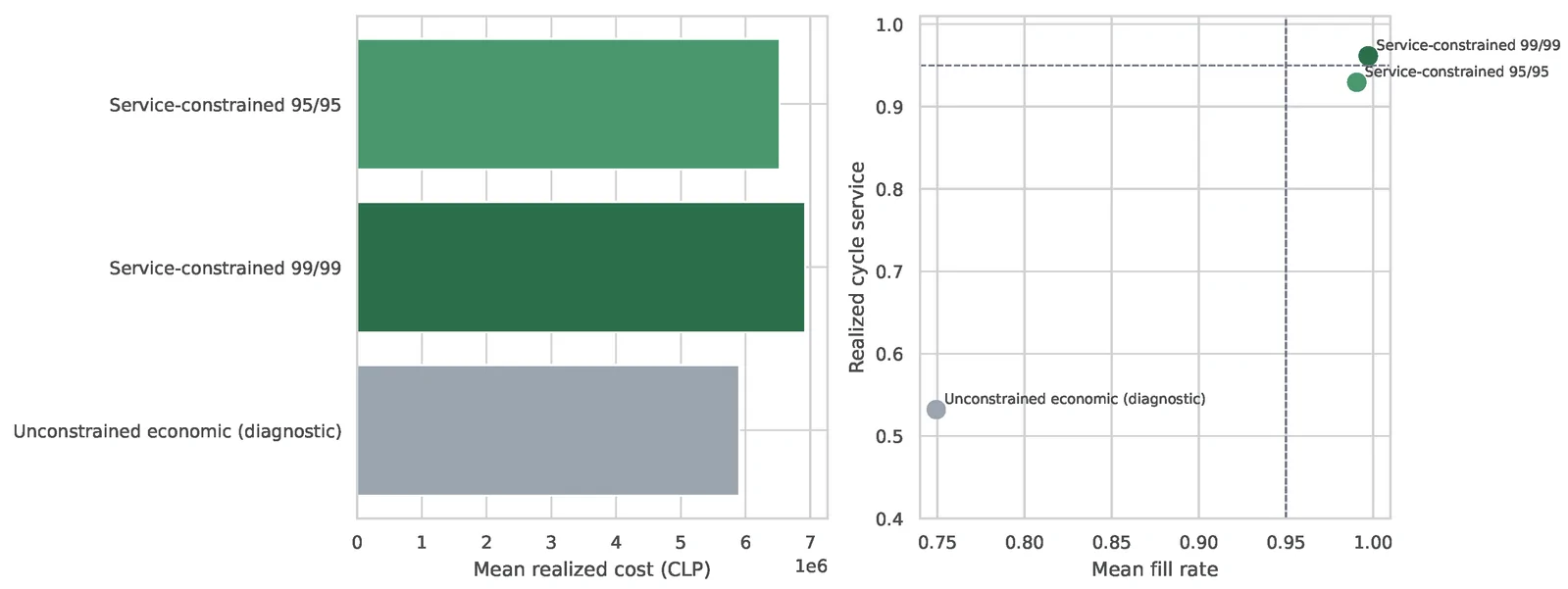
Prospective service-constrained trade-off using the selected marginal or temporal-adaptive scenario generator. SC95 and SC99 are service-qualified scenarios; the unconstrained two-stage solution is shown only as a diagnostic comparator.

**Figure 6:**
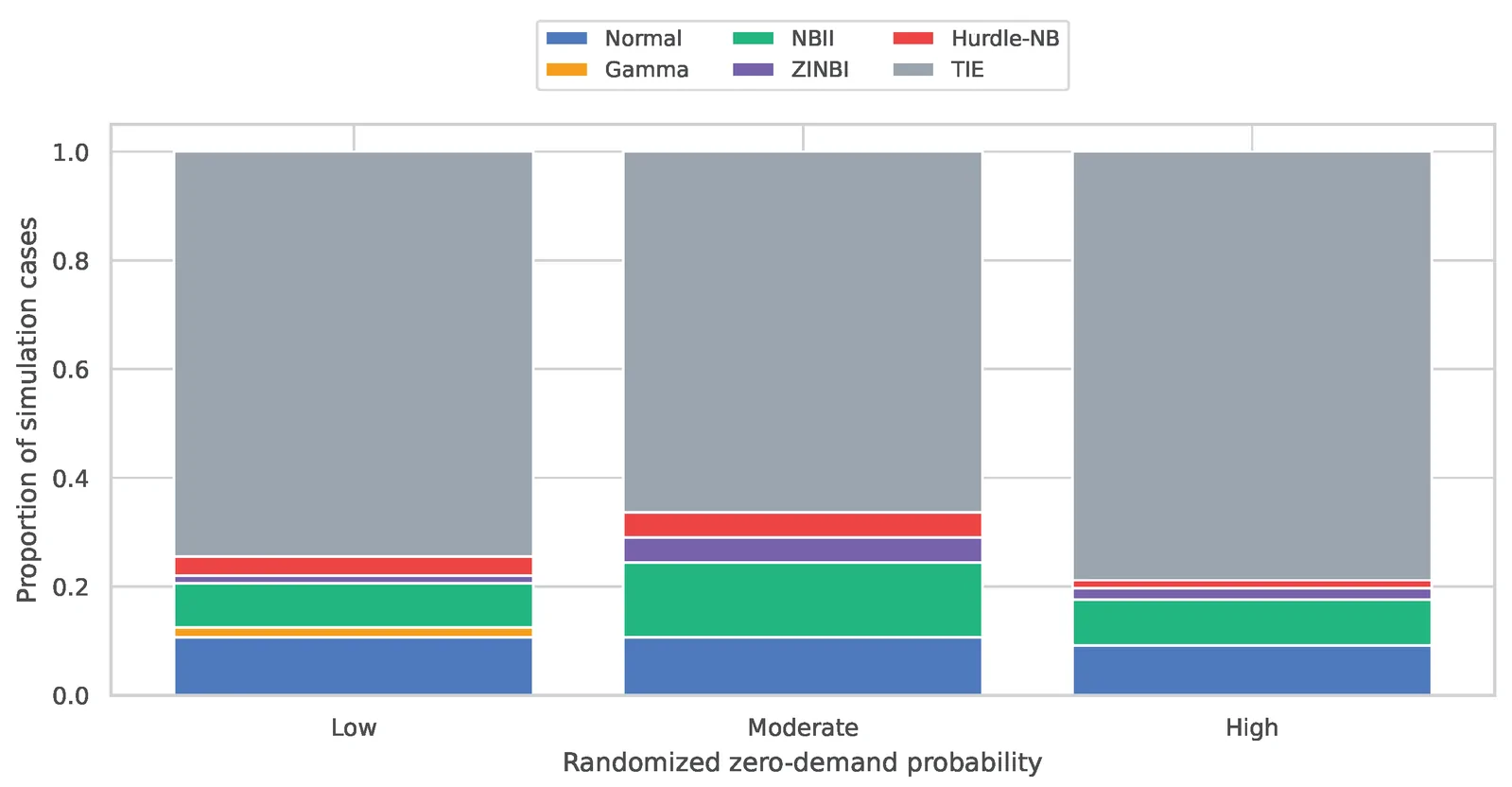
Operational winners in the diagnostic cost-loss simulation across randomized zero-demand conditions. Ties denote models within 1% of the minimum 12-period mean cost.

**Figure 7:**
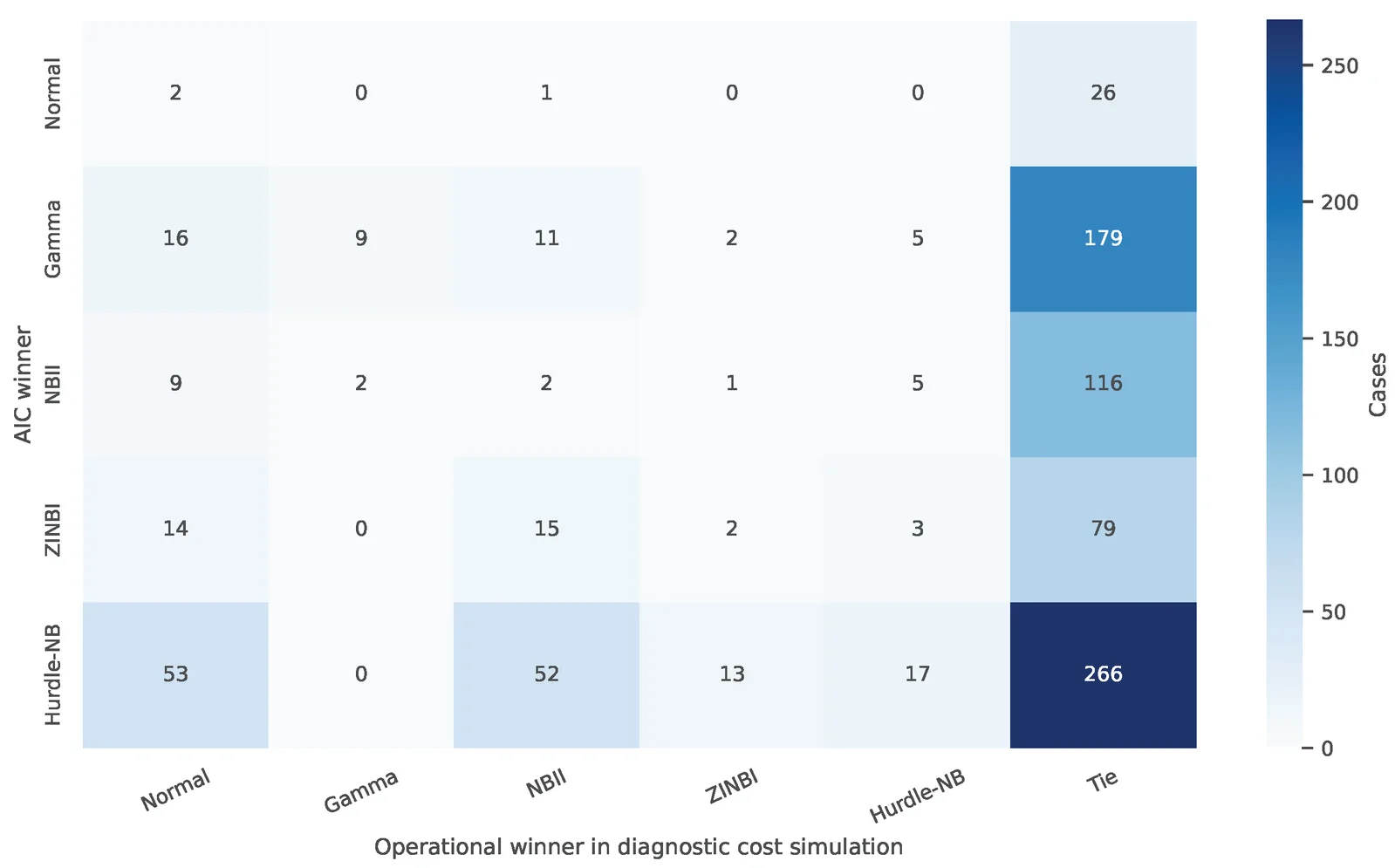
AIC winner versus operational winner in the 900-case diagnostic cost-loss simulation. Full distribution names are used in the figure; operational ties are treated as a separate class.

**Figure 8:**
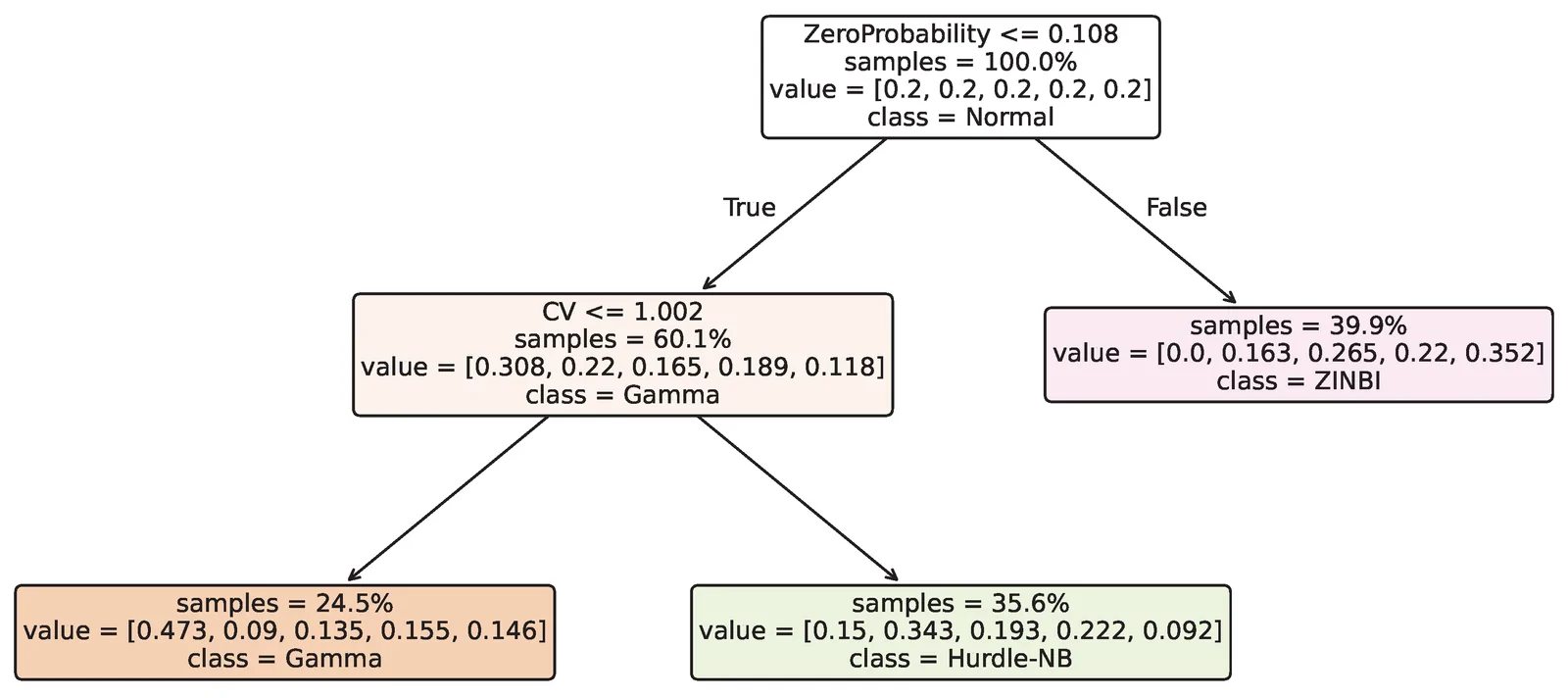
Exploratory classification tree refitted on development data after internal hyperparameter selection. Distribution classes are shown with full names; independent test performance is reported in Table 10.

**Table 1:** Positioning of the proposed service-constrained demand-profile framework relative to related literature.

Study	Setting or Problem	Main Methodological Focus	Main Contribution	Gap Addressed by This Article
Abu Zwaida et al. [4]	Hospital supply chain and drug shortages	Inventory optimization under shortage risk	Shows the importance of inventory decisions for shortage prevention	Does not connect demand-profile model selection with prospective service-constrained validation
Kelle et al. [26]	Hospital pharmacy care-unit inventory	Cost minimization subject to an α service constraint	Replaces difficult shortage-cost valuation with an explicit probability-of-no-shortage requirement	Does not compare alternative probabilistic demand families or rolling-origin model selection
Neve and Schmidt [28]	Hospital point-of-use inventory	Cost optimization with a minimum fill-rate constraint	Demonstrates service-level-constrained hospital inventory design	Focuses on inventory-record inaccuracy rather than probabilistic model selection across heterogeneous medicines
Bozkir et al. [5]	Health-network drug shortages	Stocking and transshipment under stochastic demand	Connects pharmaceutical inventory with service preservation during shortages	Focuses on inventory sharing and disruption response rather than item-level distributional selection
de Assis et al. [15]	Hospital medicines and materials	Multicriteria inventory classification	Shows that hospital inventory prioritization should extend beyond ABC	Does not compare probabilistic demand models or prospective inventory outcomes
Chen et al. [21]	Case outpatient pharmacy	Simulation optimization of inventory parameters	Demonstrates decision improvement through simulation-based stock optimization	Optimizes a selected policy rather than linking probabilistic family choice to demand profiles and service floors
Rojas et al. [30]	Pharmaceutical supply with time-dependent demand	GARMA scenario generation and two-stage stochastic programming	Shows that temporal dependence can improve scenario and lot-sizing accuracy in out-of-sample evaluation	Single-product illustration without heterogeneous zero-prone demand families, conditional portfolio-level temporal escalation, or dual service constraints
Rojas et al. [24]	Hospital pharmacy under uncertain demand	Distribution-sensitive stochastic lot sizing	Shows that demand shape can affect inventory decisions	Does not combine repeated prospective validation, dual service constraints, and model-selection screening
Pinçe et al. [17]	Intermittent-demand forecasting	Review of specialized intermittent-demand methods	Establishes the need for demand-pattern-specific forecasting approaches	Focuses on forecasting rather than hospital service-constrained inventory decisions
Rožanec and Mladenić [18]	Lumpy and intermittent demand	Occurrence–size modelling	Supports separating zero occurrence from positive demand size	Not specific to hospital medication inventory and service-level design
Li et al. [20]	Intermittent-demand forecasting	Feature-based method selection	Shows that observable demand features can guide forecasting choices	Does not translate features into service-constrained hospital replenishment decisions
This study	Public hospital pharmacy requested demand	Demand profiling; five marginal families; training-only temporal screening; conditional GLM/GARMA scenarios; two-stage SAA; dual service constraints; rolling validation	Links marginal fit and temporal dependence to scenario generation, two-stage recourse, service feasibility, and prospective calibration	Integrates heterogeneous demand modelling with conditional temporal escalation and service-constrained stochastic programming across repeated unseen months

**Table 2:** Transparent medicine-level operational inputs. Values are in 2023 Chilean pesos (CLP).

Medicine	Unit Cost (CLP)	Order Cost (CLP)	Holding/ Year	Holding/ Month	Emergency Multiplier	Historical Capacity (CLP)	December Initial Stock	December Observed Q
M01	137	20,313	14.32	1.1933	1.8714	6,718,226	2487	292,732
M02	8663	20,313	14.32	1.1933	1.8714	22,490,171	552	2596
M03	16,797	20,313	14.32	1.1933	1.8714	34,330,014	399	1525
M04	11	20,313	14.32	1.1933	1.8714	3,925,130	29,085	56,169
M05	13	20,313	14.32	1.1933	1.8714	898,743	27,841	69,134
M06	596	20,313	14.32	1.1933	1.8714	291,522	150	489
M07	322	20,313	14.32	1.1933	1.8714	138,345	660	219
M08	119	20,313	14.32	1.1933	1.8714	17,811	273	117
M09	14,637	20,313	14.32	1.1933	1.8714	2,603,951	27	84
M10	2249	20,313	14.32	1.1933	1.8714	1,228,460	330	243
M11	357	20,313	14.32	1.1933	1.8714	23,233	200	48
M12	95	20,313	14.32	1.1933	1.8714	10,317	149	41
M13	440	20,313	14.32	1.1933	1.8714	88,000	170	0

Note: The annual holding cost of CLP 14.32 per unit is converted to CLP 1.1933 per unit-month. The emergency multiplier 1.8714 represents ordinary acquisition plus the 87.14% alternative-procurement premium. December 2023 initial stock, observed Q, and historical monetary-capacity fields are retained for source documentation only and are not used as longitudinal constraints.

**Table 3:** Observed requested-demand profiles over 48 months.

Medicine	Demand Category	Mean	CV	Zero Share	Monthly Trend	Trend *p*	Annual Harmonic *p*	Lag-1 Correlation	Ljung–Box *p* (Lag 2)
M01	Recurrent-positive	205,067.1	0.126	0.000	880.89	0.001	0.440	−0.089	0.813
M02	Recurrent-positive	1952.8	0.174	0.000	−2.09	0.560	0.611	−0.181	0.370
M03	Recurrent-positive	655.3	0.478	0.000	18.55	0.000	0.732	0.859	0.000
M04	Recurrent-positive	185,245.6	0.108	0.000	700.38	0.000	0.670	0.014	0.197
M05	Recurrent-positive	70,973.3	0.116	0.000	−142.21	0.099	0.929	−0.082	0.804
M06	Intermittent	224.4	0.970	0.188	−4.81	0.032	0.922	0.076	0.820
M07	Intermittent	266.5	0.667	0.167	−2.08	0.266	0.354	−0.274	0.113
M08	Intermittent	109.4	0.645	0.042	0.82	0.267	0.586	0.131	0.644
M09	Intermittent	45.5	0.625	0.021	1.61	0.000	0.805	0.718	0.000
M10	Intermittent	182.7	1.091	0.354	1.43	0.496	0.932	−0.259	0.006
M11	Intermittent	88.3	1.157	0.417	−1.47	0.170	0.962	0.088	0.820
M12	Intermittent	48.7	1.313	0.438	−0.09	0.893	0.028	0.082	0.806
M13	Intermittent	52.7	1.795	0.688	−0.35	0.730	0.483	0.054	0.608

Note: Annual harmonic *p* is the joint F-test for 12-month sine/cosine terms after a linear time trend; it is descriptive because only four annual cycles are available. Seasonal-naive remains the prospective seasonal benchmark.

**Table 4:** Full-series distributional comparison by AIC.

Medicine	Demand Category	GA	HNB	NBII	NO	ZINBI	Winner
M01	Recurrent-positive	1119.4	1121.4	1119.4	1114.8	1121.4	NO
M02	Recurrent-positive	699.4	701.4	699.4	698.7	701.4	NO
M03	Recurrent-positive	676.9	678.9	676.9	690.9	678.9	GA
M04	Recurrent-positive	1090.9	1092.9	1090.9	1089.9	1092.9	NO
M05	Recurrent-positive	1005.1	1007.1	1005.1	1005.0	1007.1	NO
M06	Intermittent	–	560.3	597.7	656.0	560.3	ZINBI/HNB tie
M07	Intermittent	–	565.3	624.0	636.5	565.3	ZINBI/HNB tie
M08	Intermittent	–	531.7	546.3	547.8	531.7	ZINBI/HNB tie
M09	Intermittent	–	465.0	464.4	460.5	465.0	NO
M10	Intermittent	–	471.6	516.3	647.5	471.6	ZINBI/HNB tie
M11	Intermittent	–	398.1	440.6	583.4	398.1	ZINBI/HNB tie
M12	Intermittent	–	361.8	390.9	538.4	361.8	ZINBI/HNB tie
M13	Intermittent	–	245.0	270.1	576.0	245.0	ZINBI/HNB tie

Note: Abbreviations: NO = Normal; GA = Gamma; NBII = negative binomial type II; ZINBI = zero-inflated NBII; HNB = hurdle negative binomial. Gamma is not applicable when the calibration series contains zeros. When the two lowest AIC values differ by less than 0.01, the result is reported as a numerical AIC tie rather than assigning an arbitrary winner. ZINBI and HNB are alternative zero-aware parameterizations and are not interpreted causally.

**Table 5:** Selected temporal scenario models and their prospective calibration.

Medicine	Selected Temporal Origins	GLM-Trend	GARMA Origins	Marginal Coverage	Adaptive Coverage	Adaptive MAE
M01	12	0	12	0.917	0.917	11,286.9
M03	12	8	4	0.167	0.667	199.8
M04	10	10	0	1.000	1.000	11,893.6
M09	12	9	3	0.583	0.667	18.3

Note: Across all 156 rolling origins, the training-only screen detected temporal signal in 82, the Gaussian temporal branch was eligible in 72, and a temporal model ultimately replaced the marginal scenario generator in 46. The table lists only medicines with at least one selected temporal model. GARMA origins combine selected GARMA(1, 0) and GARMA(2, 0) specifications, with a linear time covariate when selected by AICc. No temporal model uses information from the held-out month.

**Table 6:** Prospective calibration before and after conditional temporal scenario modelling.

**Panel A. Central 90% predictive-interval calibration**					
**Group**	**n**	**Marginal AIC Coverage**	**Temporal-Adaptive Coverage**	**Marginal >** ***P*****95**	**Adaptive >** ***P*****95**
Overall	156	0.840	0.885	0.128	0.064
Intermittent	96	0.906	0.917	0.083	0.052
Recurrent-positive	60	0.733	0.833	0.200	0.083
Temporal-branch origins only	46	0.652	0.804	0.348	0.130
**Panel B. Scenario-expected versus realized cost after temporal adaptation**					
**Policy**	**n**	**MAPE**	**Correlation**	**Within ±10%**	
SC95	156	0.024	0.997	0.955	
SC99	156	0.007	0.999	0.974	

Note: “Marginal AIC” selects among Normal, Gamma, NBII, ZINBI, and hurdle-NB using only the training window. The temporal-adaptive framework invokes a Gaussian conditional branch only when temporal screening is positive and the response is eligible; a time-trend GLM and GARMA(1, 0)/(2, 0) candidates then compete by AICc. The branch must improve AICc by at least 2 relative to the static Gaussian model. This design extends the GLM–GARMA scenario comparison of Rojas et al. (2019) [30] to rolling hospital pharmacy decisions and service-constrained two-stage SAA.

**Table 7:** Twelve-month rolling-origin evaluation (156 medicine-month decisions per policy).

Prospective Policy	Role	n	Mean Cost (CLP)	SD Cost (CLP)	Median Cost (CLP)	Shortage Units	Overstock Units	Mean Fill Rate	Cycle Service	MAE	MASE
Service-constrained 95/95	Primary service scenario	156	6,527,589	11,120,144	1,145,007	22,044	1,025,119	0.991	0.929	2782.0	0.890
Service-constrained 99/99	High-availability scenario	156	6,921,652	11,642,693	1,232,707	14,415	1,389,641	0.997	0.962	2782.0	0.890
Unconstrained economic (diagnostic)	Diagnostic only	156	5,900,983	10,256,909	547,322	193,301	213,822	0.749	0.532	2782.0	0.890
Mean	Naive benchmark	156	6,334,265	11,100,007	452,170	422,281	123,794	0.834	0.481	3500.5	1.193
Previous	Naive benchmark	156	6,121,336	10,632,014	854,823	335,161	337,678	0.780	0.545	4313.1	1.081
Seasonal-Naive	Naive benchmark	156	6,131,690	10,672,400	747,486	227,702	128,790	0.799	0.532	2285.2	1.117

Note: At each origin only earlier months are used. Five marginal families are first compared by AIC. When the training window shows temporal structure and a Gaussian conditional response is eligible, a time-trend GLM and GARMA(1, 0)/(2, 0) candidates compete by AICc; a temporal model replaces the marginal generator only when it improves AICc by at least 2 relative to static Gaussian. The selected scenario model then feeds the two-stage SAA. SC95 and SC99 impose 95/95 and 99/99 scenario-based cycle-service/fill-rate floors; the unconstrained program is diagnostic only.

**Table 8:** Medicine-level rolling-origin cost and service outcomes.

Medicine	Profile	SC95 Cost	SC99 Cost	Diagnostic Cost	SC95 Fill	SC99 Fill	Diagnostic Fill	SC95 Cycle	SC99 Cycle	Diagnostic Cycle
M01	Recurrent-positive	34,622,790	36,250,316	31,236,046	1.000	1.000	0.985	1.000	1.000	0.583
M02	Recurrent-positive	21,895,259	23,795,605	18,175,784	1.000	1.000	0.963	1.000	1.000	0.583
M03	Recurrent-positive	20,777,652	21,278,621	21,133,871	0.957	0.980	0.851	0.583	0.750	0.250
M04	Recurrent-positive	2,567,180	2,703,396	2,368,749	0.993	0.995	0.950	0.917	0.917	0.583
M05	Recurrent-positive	1,154,149	1,233,435	962,242	1.000	1.000	0.992	1.000	1.000	0.917
M06	Intermittent	404,479	579,556	177,262	1.000	1.000	0.882	1.000	1.000	0.667
M07	Intermittent	216,505	277,561	142,014	1.000	1.000	0.830	1.000	1.000	0.583
M08	Intermittent	49,808	57,618	22,956	0.981	1.000	0.083	0.917	0.917	0.083
M09	Intermittent	1,503,837	1,632,863	1,386,422	0.995	1.000	0.905	0.833	1.000	0.500
M10	Intermittent	1,334,654	1,718,344	1,023,568	0.952	0.989	0.633	0.833	0.917	0.500
M11	Intermittent	123,811	165,242	61,242	1.000	1.000	0.417	1.000	1.000	0.417
M12	Intermittent	37,356	45,204	8208	1.000	1.000	0.417	1.000	1.000	0.417
M13	Intermittent	171,180	243,719	14,410	1.000	1.000	0.833	1.000	1.000	0.833

Note: SC95 and SC99 solve the same two-stage sample-average program subject to explicit scenario-based service floors. The unconstrained two-stage program is retained only as a diagnostic comparator. Costs are mean realized CLP per medicine-month.

**Table 9:** Randomized simulation design, statistical–operational agreement, and diagnostic operational consequences.

Metric	Value
Simulation cases	900
SAA scenarios per candidate fit	256
Future evaluation periods per case	12
Operational ties (within 1%)	666
Unique operational winners	234
AIC/operational agreement among unique winners	0.137
Mean AIC-selected cost regret (%)	2.19%
Median AIC-selected cost regret (%)	0.11%
Mean AIC-selected shortage rate	0.564
Mean AIC-selected overstock rate	0.317
Mean AIC-selected fill rate	0.436

Note: Each of the 900 randomized cases fits the eligible marginal candidates on 48 periods, generates 256 SAA demand scenarios per candidate fit, and evaluates the resulting unconstrained two-stage target over 12 new periods. Operational ties are declared when all models within the tied set are no more than 1% above the minimum 12-period mean cost. AIC-selected cost regret is relative to the minimum fitted-model cost in the same case; shortage and overstock rates are normalized by total future demand. These diagnostic simulation outcomes are not service-qualified hospital policies.

**Table 10:** Independent-test performance of the exploratory decision tree.

Development n	Test n	Depth	Minimum Leaf	Test Accuracy	Balanced Accuracy	Rare Winner Cases Excluded
163.000	71.000	2.000	40.000	0.056	0.213	0.000

Note: Depth and minimum leaf size are selected using development data only; the held-out test set is not used for tuning.

## Data Availability

The anonymized data, complete rolling-origin marginal and temporal model selections, scenario-calibration outputs, two-stage stochastic-programming results, randomized simulation microdata, analysis scripts, generated tables and figures, and the reproducibility manifest supporting this study are publicly available in Zenodo at DOI: 10.5281/zenodo.21456322. The repository contains the source CSV files and Python 3.13 routines required to reproduce the empirical and simulation analyses reported in this article.

## References

[1] Franco C., Alfonso-Lizarazo E. (2020). Optimization under Uncertainty of the Pharmaceutical Supply Chain in Hospitals. Comput. Chem. Eng..

[2] Baumer A.M., Clark A.M., Witmer D.R., Geize S.B., Vermeulen L.C., Deffenbaugh J.H. (2004). National Survey of the Impact of Drug Shortages in Acute Care Hospitals. Am. J. Health-Syst. Pharm..

[3] Maestre-Torreblanca J.M., Isla-Tejera B., Fernández-García M.I., del Prado-Llergo J.R., Álamo-Cantarero T., Fernández-Camacho E. (2012). Stockout Risk Analysis and Minimization Applied to Hospital Pharmacy Management. Farm. Hosp..

[4] Abu Zwaida T., Pham C., Beauregard Y. (2021). Optimization of Inventory Management to Prevent Drug Shortages in the Hospital Supply Chain. Appl. Sci..

[5] Bozkir C.D.C., Kundakcioglu O.E., Henry A.C. (2022). Hospital Service Levels during Drug Shortages: Stocking and Transshipment Policies for Pharmaceutical Inventory. J. Glob. Optim..

[6] Tucker E.L., Daskin M.S. (2022). Pharmaceutical Supply Chain Reliability and Effects on Drug Shortages. Comput. Ind. Eng..

[7] American Society of Health-System Pharmacists Drug Shortages Statistics: National Drug Shortages. January 2001 to June 2026. https://www.ashp.org/drug-shortages/shortage-resources/drug-shortages-statistics.

[8] Fallahnezhad M., Langarizadeh M., Vahabzadeh A. (2024). Key Performance Indicators of Hospital Supply Chain: A Systematic Review. BMC Health Serv. Res..

[9] Yap W.S., Cheong M.W.L., Yeap L.L. (2026). Pharmacy Inventory Management Practices and Constraints in Asia-Pacific Hospitals: A Systematic Review and Qualitative Synthesis. J. Pharm. Policy Pract..

[10] Devnani M., Gupta A.K., Nigah R. (2010). ABC and VED Analysis of the Pharmacy Store of a Tertiary Care Teaching, Research and Referral Healthcare Institute of India. J. Young Pharm..

[11] Kant S., Haldar P., Singh A., Kankaria A. (1997). Inventory Management in Medical Stores by ABC Analysis and VED Analysis. J. Acad. Hosp. Adm..

[12] Pund S.B., Kuril B.M., Doibale M.K., Ankushe R.T., Kumar P., Siddiqui N. (2016). ABC-VED Matrix Analysis of Government Medical College, Aurangabad Drug Store. Int. J. Community Med. Public Health.

[13] Ramanathan R. (2006). ABC Inventory Classification with Multiple-Criteria Using Weighted Linear Optimization. Comput. Oper. Res..

[14] Axsäter S. (2015). Inventory Control.

[15] de Assis A.G., dos Santos A.F.A., dos Santos L.A., da Costa J.F., Cabral M.A.L., de Souza R.P. (2022). Classification of Medicines and Materials in Hospital Inventory Management: A Multi-Criteria Analysis. BMC Med. Inform. Decis. Mak..

[16] Syntetos A.A., Boylan J.E., Croston J.D. (2005). On the Categorization of Demand Patterns. J. Oper. Res. Soc..

[17] Pinçe Ç., Turrini L., Meissner J. (2021). Intermittent Demand Forecasting for Spare Parts: A Critical Review. Omega.

[18] Rožanec J.M., Mladenić D. (2022). Reframing Demand Forecasting: A Two-Fold Approach for Lumpy and Intermittent Demand. Sustainability.

[19] Sanguri K., Shukla N., Tiwari M.K. (2021). Forecasting of Intermittent Demands under the Risk of Inventory Obsolescence. J. Forecast..

[20] Li L., Kang Y., Petropoulos F., Li F. (2023). Feature-Based Intermittent Demand Forecast Combinations: Accuracy and Inventory Implications. Int. J. Prod. Res..

[21] Chen C.N., Lai C.H., Lu G.W., Huang C.C., Wu L.J., Lin H.C., Chen P.S. (2022). Applying Simulation Optimization to Minimize Drug Inventory Costs: A Study of a Case Outpatient Pharmacy. Healthcare.

[22] Robert L., Cuvelier E., Rousselière C., Gautier S., Odou P., Beuscart J.B., Décaudin B. (2023). Detection of Drug-Related Problems through a Clinical Decision Support System Used by a Clinical Pharmacy Team. Healthcare.

[23] Orsini F.F., Bellavia D., Schettini F., Foglia E. (2025). The Impact of Automation and Digitalization in Hospital Medication Management: Economic Analysis in the European Countries. Healthcare.

[24] Rojas F., Leiva V., Huerta M., Martin-Barreiro C. (2021). Lot-Size Models with Uncertain Demand Considering Its Skewness/Kurtosis and Stochastic Programming Applied to Hospital Pharmacy with Sensor-Related COVID-19 Data. Sensors.

[25] Shapiro A., Dentcheva D., Ruszczyński A. (2009). Lectures on Stochastic Programming: Modeling and Theory.

[26] Kelle P., Woosley J., Schneider H. (2012). Pharmaceutical Supply Chain Specifics and Inventory Solutions for a Hospital Case. Oper. Res. Health Care.

[27] Uthayakumar R., Priyan S. (2013). Pharmaceutical Supply Chain and Inventory Management Strategies: Optimization for a Pharmaceutical Company and a Hospital. Oper. Res. Health Care.

[28] Neve B.V., Schmidt C.P. (2022). Point-of-Use Hospital Inventory Management with Inaccurate Usage Capture. Health Care Manag. Sci..

[29] Benjamin M.A., Rigby R.A., Stasinopoulos D.M. (2003). Generalized Autoregressive Moving Average Models. J. Am. Stat. Assoc..

[30] Rojas F., Leiva V., Wanke P., Lillo C., Pascual J. (2019). Modeling Lot-Size with Time-Dependent Demand Based on Stochastic Programming and Case Study of Drug Supply in Chile. PLoS ONE.

[31] Akaike H. (1974). A New Look at the Statistical Model Identification. IEEE Trans. Autom. Control.

[32] Hurvich C.M., Tsai C.L. (1989). Regression and Time Series Model Selection in Small Samples. Biometrika.

[33] Cheng L., Tsou C.S., Yang D.Y. (2015). Cost-Service Tradeoff Analysis of Reorder-Point-Lot-Size Inventory Models. J. Manuf. Syst..

[34] Tyworth J.E., O’Neill L. (1997). Robustness of the Normal Approximation of Lead-Time Demand in a Distribution Setting. Nav. Res. Logist..

[35] Burgin T.A. (1975). The Gamma Distribution and Inventory Control. Oper. Res. Q..

[36] Cameron A.C., Trivedi P.K. (1986). Econometric Models Based on Count Data: Comparisons and Applications of Some Estimators and Tests. J. Appl. Econom..

[37] Hilbe J.M. (2011). Negative Binomial Regression.

[38] Lambert D. (1992). Zero-Inflated Poisson Regression, with an Application to Defects in Manufacturing. Technometrics.

[39] Mwalili S.M., Lesaffre E., Declerck D. (2008). The Zero-Inflated Negative Binomial Regression Model with Correction for Misclassification: An Example in Caries Research. Stat. Methods Med. Res..

[40] Mullahy J. (1986). Specification and Testing of Some Modified Count Data Models. J. Econom..

[41] Stasinopoulos D.M., Rigby R.A. (2007). Generalized Additive Models for Location Scale and Shape (GAMLSS) in R. J. Stat. Softw..

[42] Breiman L., Friedman J.H., Olshen R.A., Stone C.J. (1984). Classification and Regression Trees.

[43] Rojas F. (2026). Dataset and Code: A Data-Driven Decision Support Framework for Hospital Pharmacy Medication Supply Under Demand Uncertainty.

